# Multifaceted Intervention to Prevent Venous Thromboembolism in Patients Hospitalized for Acute Medical Illness: A Multicenter Cluster-Randomized Trial

**DOI:** 10.1371/journal.pone.0154832

**Published:** 2016-05-26

**Authors:** Pierre-Marie Roy, Antoine Rachas, Guy Meyer, Grégoire Le Gal, Pierre Durieux, Dominique El Kouri, Didier Honnart, Jeannot Schmidt, Catherine Legall, Pierre Hausfater, Jean-Marie Chrétien, Dominique Mottier, PREVENU study group

**Affiliations:** 1 Département de Médecine d’Urgence, Centre Vasculaire et de la Coagulation, CHU Angers, Institut MITOVASC, EA3860, Université d’Angers, Angers, France; 2 Unité d’Épidémiologie et de Recherche Clinique, Assistance Publique Hôpitaux de Paris, Hôpital Européen Georges Pompidou, Centre d’investigation Épidémiologique 4, INSERM, Paris, France; 3 Service de Pneumologie et Soins Intensifs, Hôpital Européen Georges Pompidou, Assistance Publique Hôpitaux de Paris, Université Paris Descartes, Sorbonne Paris Cité, INSERM U 970, CIC 1418, Paris, France; 4 Département de Médecine Interne et Pneumologie, CHU de la Cavale Blanche, Université de Bretagne Occidentale, EA3878 (GETBO), CIC INSERM 1412, Brest, France; 5 Département de Santé Publique et Informatique Médicale, Hôpital Européen Georges Pompidou, Assistance Publique Hôpitaux de Paris, Université Paris Descartes, Sorbonne Paris Cité, INSERM UMRS 872, Centre de recherche des Cordeliers, Paris, France; 6 Service des Urgences, Médecine Polyvalente, CHU Hôtel Dieu, Nantes, France; 7 Département de Médecine d’Urgence, CHU Dijon, Hôpital du Bocage, Dijon, France; 8 Service d’Accueil des Urgences, CHU Gabriel Montpied, Université de Clermont-Ferrand, Clermont-Ferrand, France; 9 Service des Urgences, CH Argenteuil Victor Dupouy, Argenteuil, France; 10 Service des Urgences, CHU La Pitié Salpétrière, Assistance Publique Hôpitaux de Paris, Université Paris 06 UPMC, Paris, France; 11 Délégation à la recherche clinique et innovation, cellule de gestion des données et évaluation, CHU Angers, Angers, France; 12 Département de Médecine Interne et Pneumologie, CHU de la Cavale Blanche, Université de Bretagne Occidentale, EA3878 (GETBO), CIC INSERM 1412, Brest, France; Maastricht University Medical Center, NETHERLANDS

## Abstract

**Background:**

Misuse of thromboprophylaxis may increase preventable complications for hospitalized medical patients.

**Objectives:**

To assess the net clinical benefit of a multifaceted intervention in emergency wards (educational lectures, posters, pocket cards, computerized clinical decision support systems and, where feasible, electronic reminders) for the prevention of venous thromboembolism.

**Patients/Methods:**

Prospective cluster-randomized trial in 27 hospitals. After a pre-intervention period, centers were randomized as either intervention (n = 13) or control (n = 14). All patients over 40 years old, admitted to the emergency room, and hospitalized in a medical ward were included, totaling 1,402 (712 intervention and 690 control) and 15,351 (8,359 intervention and 6,992 control) in the pre-intervention and intervention periods, respectively.

**Results:**

Symptomatic venous thromboembolism or major bleeding (primary outcome) occurred at 3 months in 3.1% and 3.2% of patients in the intervention and control groups, respectively (adjusted odds ratio: 1.02 [95% confidence interval: 0.78–1.34]). The rates of thromboembolism (1.9% *vs*. 1.9%), major bleedings (1.2% *vs*. 1.3%), and mortality (11.3% *vs*. 11.1%) did not differ between the groups. Between the pre-intervention and intervention periods, the proportion of patients who received prophylactic anticoagulant treatment more steeply increased in the intervention group (from 35.0% to 48.2%: +13.2%) than the control (40.7% to 44.1%: +3.4%), while the rate of adequate thromboprophylaxis remained stable in both groups (52.4% to 50.9%: -1.5%; 49.1% to 48.8%: -0.3%).

**Conclusions:**

Our intervention neither improved adequate prophylaxis nor reduced the rates of clinical events. New strategies are required to improve thromboembolism prevention for hospitalized medical patients.

**Trial Registration:**

ClinicalTrials.gov NCT01212393

## Introduction

Hospitalization for acute medical illness is a significant risk factor of venous thromboembolism (VTE), accounting for up to 20% of all VTEs and 80% of in-hospital fatal cases of pulmonary embolism.[1–3] Pharmacological regimens have been shown to reduce thromboembolic events[4, 5] and for over a decade, consensus guidelines have recommended thromboprophylaxis for high-risk medical patients.

Yet prophylaxis appears to be used inappropriately and often underused for hospitalized medical patients.[6, 7] As system-wide standardized interventions may be more effective than relying on individual physicians’ routine practices, the American College of Chest Physicians has recommended “for every hospital, that a formal active strategy addressing the prevention of VTE be developed”.[5] A recent meta-analysis has suggested that alerts or multifaceted interventions increase prophylaxis prescription, although how this finding applies to a VTE or bleeding setting remains unknown, given that most studies are underpowered to assess these outcomes.[8]

We hypothesized that a multifaceted intervention for VTE guidelines implementation in emergency departments should improve prophylaxis use for patients hospitalized in medical wards and decrease the rate of VTE or major bleedings.

## Methods

### Design

We performed a multicenter prospective controlled cluster-randomized trial in community and academic hospitals.

### Centers

Hospitals were eligible for participation if the annual number of visits in their emergency department was >30,000.

### Patients

We prospectively enrolled all consecutive patients over 40 years old presenting to the emergency department of the participating sites for acute medical illness and requiring hospitalization in a medical ward.

Patients were excluded from analysis if they were hospitalized for less than 48 hours, if venous thrombosis or pulmonary embolism was diagnosed within 48 hours following admission (in order to rule out any VTE that had occurred before hospital admission), and if they received anticoagulant treatment at a therapeutic dosage for another reason than acute VTE at the admission and/or during hospitalization.

### Pre-intervention period

All included emergency departments participated in a 1-week observational pre-intervention period. During this period, while information was collected on thromboprophylaxis use, patients were not followed-up for 3 months. This pre-intervention period was aimed at obtaining baseline values for comparison with the intervention period.

### Intervention period

A random number table was used to assign hospitals to either the intervention or standard practice group (control group). Randomization was stratified in order to include the same number of academic hospitals and centers using a computerized medical file in each group.

### Implementation of recommendations in the intervention group

The intervention was multifaceted, based on educational lectures, posters, and pocket cards, computerized clinical decision support systems, and computerized reminders (S1 and S2 Posters).

Emergency physicians and residents attended a lecture that presented the guidelines for thromboprophylaxis use in acutely ill medical patients.[5, 9] In summary, thromboprophylactic treatment was recommended for high-risk patients, i.e. patients confined to bed presenting with an acute medical condition associated with a high risk of VTE, and patients presenting with an acute medical condition associated with an intermediate risk of VTE and, at least, one VTE risk factor (Table 1). In patients for whom prophylaxis was recommended but with contraindication to antithrombotic treatment, a mechanical prophylaxis method was recommended, namely by means of compression stockings or intermittent pneumatic compression devices. The key objectives were to systematically evaluate the risk of thrombosis and to prescribe and initiate venous thromboembolism prophylaxis as soon as possible in those at high risk (Table 1).

**Table 1:** Definition of high-risk patients and recommendations of thromboprophylactic treatment.

High risk of venous thromboembolism (VTE) acute medical condition*	
Congestive heart failure (New York Heart Association class III or IV)	
Acute respiratory failure	
Recent ischemic stroke or neurological injury with lower extremity weakness (< 30 days)	
Recent myocardial infarct or acute coronary syndrome (< 30 days)	
**Intermediate risk of VTE acute medical condition** § **and VTE risk factor(s)** †	
Sepsis	Age ≥ 75 years
Acute rheumatic disorder	Previous VTE
Acute inflammatory disease	Active cancer
	Obesity (body mass index ≥ 30)
	Varicose veins
	Hormone therapy (anti-androgen or estrogen)
	Chronic heart or respiratory failure
	Pregnancy or recent post-partum (< 30 days)

Thromboprophylaxis was recommended for the following patients confined to bed:

i) Patients with a high risk of venous thromboembolism acute medical condition *

ii) Patients with an intermediate risk of VTE acute medical condition §, if they had at least one additional VTE risk factor †

The indications for thromboprophylaxis in hospitalized medical patients were summarized on posters and pocket cards. The list of antithrombotic treatments approved for this indication and their recommended doses were also provided.[5, 9]

A computer-based clinical decision support system was developed and installed on the emergency departments’ computers in the intervention group. According to patient diagnosis and comorbidities, this system provided information on whether or not thromboprophylaxis was recommended, and, should this be the case, indicated the adequate treatment and doses.

In the emergency wards with access to computerized medical files, we planned to implement a software program which would systematically remind the consulting physician to assess thrombosis risk and start prophylaxis in patients hospitalized in a medical setting.

The intervention period lasted for 8 to 10 weeks.

### Control group

No intervention was performed in the centers allocated to the control group. The principal investigators were instructed to continue their practice as usual.

### Clinical outcomes

The primary outcome was defined at the patient level as a composite endpoint comprising of symptomatic VTE events and major bleedings during the three months following hospital admission. VTE events, major bleedings, and all-cause mortality during the 3 months following admission and during hospitalization were secondary outcomes.

VTE was defined as one of the following events: i) deep vein thrombosis, ii) pulmonary embolism, and iii) sudden death with no obvious cause (possible fatal pulmonary embolism). The VTE events had to be symptomatic and confirmed by objective tests.[10, 11] Major bleeding was defined according to the International Society on Thrombosis and Haemostasis’ criteria (any bleeding resulting in death, in a critical organ or resulting in the transfusion of at least two packs of blood red cells).[12] For patients who experienced several events, *i*.*e*. of venous thromboembolism and/or major bleedings, only the first was taken into account.

An independent adjudication committee, blinded to the group allocation, performed a chart review and analyzed all suspected outcome events.

### Prophylaxis adequacy

An adequacy assessment was performed, which was blinded to group assignment and mostly automated, based on several criteria:

- Patient’s risk of VTE (high-risk or low-risk) according to the main reason for admission and VTE risk factors (Table 1)
- Delay between admission and treatment initiation (< 5 days)
- Duration of anticoagulation (at least 5 days or until discharge)
- Eventual contraindication for anticoagulant treatment, especially bleeding
- Dosage of anticoagulant treatment according to recommendations.

Thromboprophylaxis was considered as adequate if: i) for high-risk patients with thromboprophylaxis recommended as per the guidelines, antithrombotic treatment was initiated before Day 5 and administered at the correct dosage for at least 5 days or ii) for low-risk patients with no indication or those with contra-indication for anticoagulant treatment, antithrombotic treatment was not administered [5, 9]. Mechanical thromboprophylaxis was not assessed.

As thromboembolism could occur very early after admission [3] and in order to assess the rate of thromboprophylactic treatment prescribed by the emergency physicians, we performed a sensitivity analysis considered as adequate for high-risk patients, antithrombotic treatment initiated within the first 2 days following admission.

### Sample size

With the study design assuming 30 participating centers divided into two groups of 15, an intra-cluster correlation of 0.01, a 5% combined rate of VTE or major bleedings in the control group, a total of 16,170 patients with 8,085 in each group were required to detect a 1.5 percentage absolute difference between the two groups, with 3.5% in the intervention group, at a power of 80% and significance level of 5%.[13]

Considering that approximately 15% of patients would be hospitalized for less than 48 hours and 5% would be lost to follow-up, we planned to enroll a total of 20,000 patients.

### Statistical analysis

The statistical analysis plan was defined following closure of the database, prior to any statistical analysis (S1 Statistical analysis plan). The statistician was blinded to the randomization group and center names. We estimated the odds ratio in mixed-effects logistic regression, adjusting for significant patient- and center-level confounders and taking into account the dependence between patients from the same hospital, also known as the clustering effect.[14]

Subgroup analyses were conducted for age (≤ or >75 years old) and according to whether or not a treatment was recommended. Sensitivity analyses were conducted using other definitions of the primary endpoint, not considering unexplained sudden deaths, such as venous thromboembolism events, and considering all unexplained deaths as venous thromboembolism events.

The change in practice adequacy was compared between the pre-intervention and intervention periods by using a mixed-effects logistic regression model, including study group, study period, and a term for the group-by-period interaction, adjusting for significant confounders. Adjusted absolute differences from the marginal predictions of probabilities in the two groups were derived from the logistic equation. P-values and 95% confidence intervals (95% CIs) were computed by using the standard errors estimated with the delta method. In order to compare our results to others, we performed a post-hoc analysis of the rate of thromboprophylactic treatment regardless of adequacy to recommendations.

We estimated intra-class correlation by using the Murray formula.[15] All statistical analyses were performed using the STATA software (release 11; Stata Corp., College Station, Texas, USA).

### Ethics

The Ethics Committee of Angers University Hospital approved this study for all centers. The study was registered and approved by the French competent authorities on June 04, 2009 prior to perform the first inclusion (n°: 09–256). French regulations consider randomized cluster trials aiming to improve the implementation of good practice recommendations as non-interventional trials and do not require written consent from participants. However, we sought oral consent from patients for follow-up and informed all patients of their right to request the withdrawal of their personal data. The study was also registered at the international registry of clinical trials on September 29, 2010 (ClinicalTrials.gov Identifier: NCT01212393).

## Results

### Clusters

**Fig 1:**
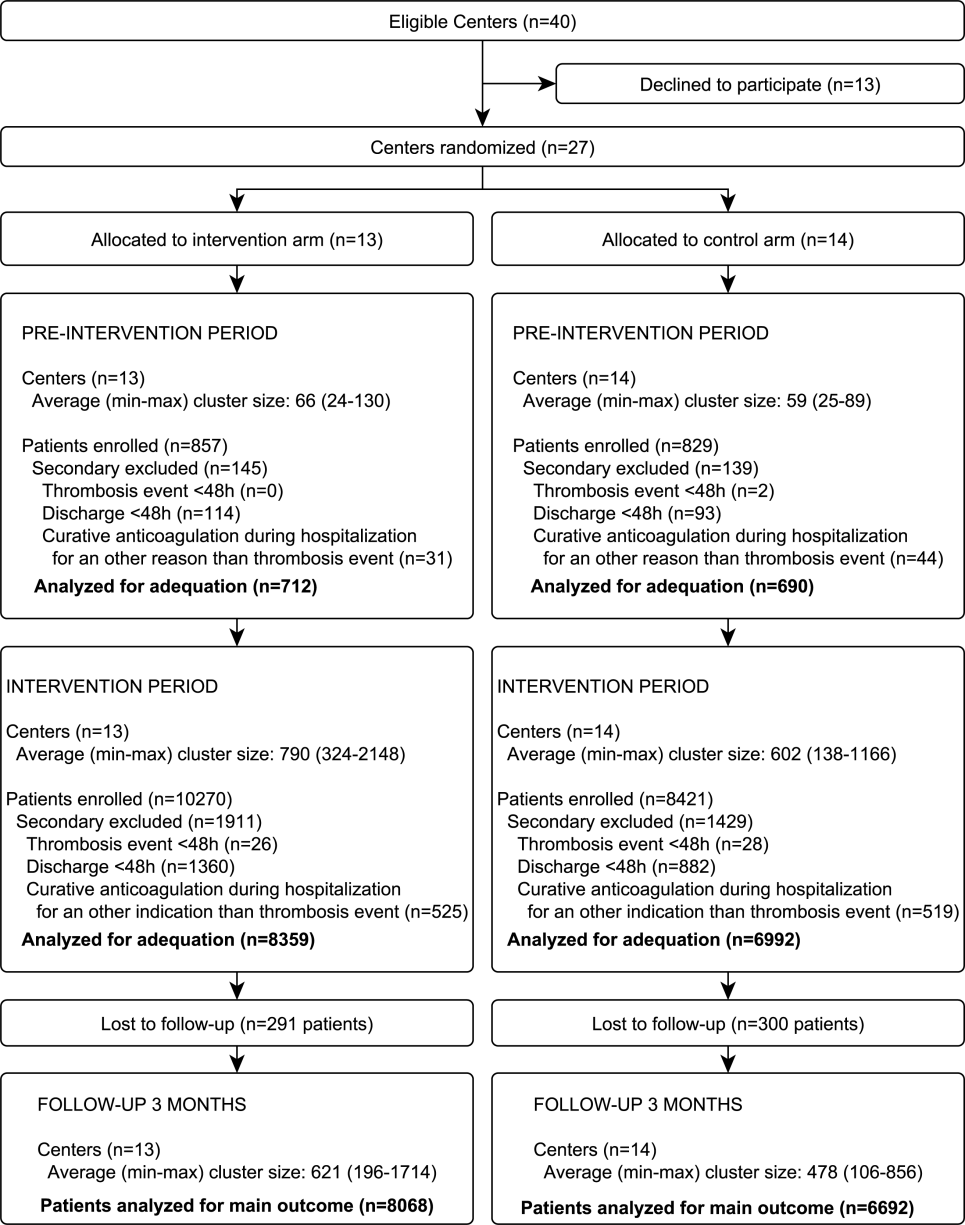
Flow chart.

In total, 40 hospitals were screened for eligibility, six refused participation, and seven could not participate for logistical reasons (Fig 1). The 27 participating centers were all located throughout France and included 20 academic and seven community hospitals. All completed the study between November 2009 for the first center and November 2010 for the last center.

At the end of the pre-intervention period, the centers were randomized into the intervention group (n = 13) or control group (n = 14). Ten centers were academic hospitals and seven centers used computerized medical files, in each group.

In each of the intervention group centers, educational lectures were organized and posters, pocket cards, and a downloadable computerized clinical decision support system were provided. However, for technical and administrative reasons, computerized reminders could be implemented in only two centers.

### Patients

A total of 20,377 patients were enrolled, 1,686 in the pre-intervention period and 18,691 in the intervention period. Of these, 284 (16.8%) and 3,340 patients (17.9%) were secondarily excluded primarily due to early discharge, in the pre-intervention and intervention periods, respectively, leaving 1,402 (712 in the intervention group and 690 in the control group) and 15,351 (8,359 intervention and 6,992 control) patients in each period, respectively. The rate of patients lost to follow-up was 3.9%, resulting in a total of 14,760 patients analyzed for the primary outcome, composed of 8,068 in the intervention group and 6,692 in the control group (Fig 1).

All data regarding demographic characteristics, past medical history and co-morbidities, medication on admission, main acute medical condition (principal reason for hospitalization), procedures performed during hospitalization, and length of hospital stay has been provided in Table 2 and S1 Table.

**Table 2:** Characteristics of the study population.

	Intervention group		Control group	
	Period		Period	
	Pre-intervention	Intervention	Pre-intervention	Intervention
	N = 712	N = 8359	N = 690	N = 6992
Demographic characteristics				
Median age—y (IQR)	73 (58–83)	74 (59–83)	72 (59–81)	73 (58–82)
Male—n (%)	361 (50.7)	4136 (49.5)	352 (51.0)	3536 (50.6)
Patient history & co-morbidities				
Previous thromboembolism—n (%)	38 (5.3)	487 (5.8)	45 (6.5)	448 (6.4)
Chronic respiratory disease—n (%)	112 (15.8)	1335 (16.0)	115 (16.7)	1226 (17.6)
Congestive heart failure—n (%)	66 (9.3)	849 (10.2)	87 (12.6)	1038 (14.9)
Chronic inflammatory disease—n (%)	33 (4.7)	293 (3.5)	32 (4.7)	263 (3.8)
Active malignant condition—n (%)	111 (15.6)	1219 (14.6)	95 (13.8)	1021 (14.6)
Surgery within 1 month—n (%)	13 (1.8)	176 (2.1)	20 (2.9)	113 (1.6)
Hospitalization within 1 month—no. (%)	69 (9.7)	753 (9.0)	76 (11.0)	676 (9.7)
Renal function at admission				
Median creatinin—μmol/l (IQR)	80 (63–105)	78 (64–103)	88 (69–117)	86 (69–113)
Creatinin clearance* < 30ml/min–n (%)	55 (8.3)	478 (7.2)	53 (7.0)	565 (8.9)
Main acute medical condition				
With high risk of thromboembolic event **— n (%)	208 (29.3)	2555 (30.7)	197 (28.7)	2292 (33.0)
With intermediate risk** —n (%)	172 (24.2)	2382 (28.7)	188 (27.4)	1916 (27.5)
With low risk** —n (%)	302 (42.5)	3037 (36.5)	276 (40.2)	2437 (35.0)
Acute or recent bleeding—n (%)	28 (3.9)	336 (4.0)	25 (3.6)	310 (4.5)
Procedures during hospitalization				
Surgery (general or regional anesthesia)—n (%)	15 (2.1)	253 (3.4)	36 (5.2)	320 (4.6)
Indwelling central venous catheter—n (%)	9 (1.3)	73 (0.9)	12 (1.7)	90 (1.3)
Duration of hospitalization				
Median—days (IQR)	7 (4–12)	7 (4–13)	8 (4–14)	8 (4–14)
>14 days—n (%)	131 (18.4)	1644 (19.7)	166 (24.1)	1625 (23.2)

* Creatinin clearance calculated by simplified MDRD formula, considering all patients were white

** Risk stratification based on the 2008 American College of Chest Physicians guidelines for thromboprophylaxis in acutely ill medical patients. IQR: interquartile range.

### Primary and secondary clinical outcomes

The primary outcome occurred in 250 of 8,359 patients (3.1%) in the intervention group and in 214 of 6,992 (3.2%) in the control group (adjusted odds ratio: 1.02; 95% CI: 0.78–1.34; p = 0.87) (Table 3). Thromboembolic events (1.9% *vs*. 1.9%), major bleedings (1.2% *vs*. 1.3%), and all-cause mortality (11.3% *vs*. 11.1%) did not differ between the groups at three months.

**Table 3:** Clinical events.

	Intervention group	Control group	OR (95% CI) adjusted for cluster effect only*	p	OR (95% CI) adjusted for cluster and fixed effects**	p
Thromboembolic event or major bleeding—no./N (%)	250/8068 (3.1)	214/6692 (3.2)	0.99 (0.75–1.32)	0.97	1.02 (0.78–1.34)	0.87
First event:						
Thromboembolic event—no./N (%)	150/8068 (1.9)	128/6692 (1.9)	1.02 (0.70–1.47)	0.94	1.01 (0.71–1.45)	0.93
Pulmonary embolism (including fatal)—no.	31	36				
Unexplained sudden death—no.	71	58				
Proximal deep vein thrombosis—no.	24	22				
Distal deep vein thrombosis—no.	24	12				
Major bleeding (including fatal)—no./N (%)	100/8068 (1.2)	86/6692 (1.3)	0.93 (0.57–1.50)	0.76	0.99 (0.60–1.63)	0.97
Non-fatal major bleeding—no.	76	74				
Fatal bleeding—no.	24	12				
Death—no./N (%)	940/8298 (11.3)	764/6884 (11.1)	0.93 (0.72–1.19)	0.57	0.96 (0.75–1.23)	0.75
Fatal pulmonary embolism—no.	3	5				
Unexplained sudden death—no.	72	59				
Fatal hemorrhage—no.	37	25				
Death unrelated to PE or hemorrhage—no.	663	534				
Death with insufficient information—no.	165	141				

*OR from mixed logistic regression including center as random intercept

** Fixed effects were:—for thromboembolic event and major bleeding: age, gender, history of active malignant condition, hospitalization within 1 month, renal function at admission, main acute medical condition, surgery (general or regional anesthesia), indwelling central venous catheter or cardiac stimulator implantation, length of hospitalization, university hospital;—for mortality: same factors, plus history of previous thromboembolism, history of congestive heart failure, antiplatelet therapy; OR: odds ratio; CI: confidence interval; PE: pulmonary embolism

There was no difference regarding thromboembolic events or major bleedings between the two arms in patients aged ≤75 years or >75 years, nor between those with or without an indication for prophylaxis. The use of different definitions of VTE did not change the results. The in-hospital event rates did not differ between the groups (S2, S3 and S4 Tables).

### Prophylactic practice outcomes

During the pre-intervention period, the rate of adequate thromboprophylaxis practice was 52.4% in the intervention group and 49.1% in the control group. During the intervention period, the rate of adequate practice did not change significantly in either group (-1.5% in the intervention group and -0.3% in the control group) (Table 4). Similar results were produced if the thromboprophylaxis was considered adequate only if the treatment was initiated within the first 2 days, as opposed to 5, or, in the subgroup analysis in terms of whether an antithrombotic treatment was indicated (S5 and S6 Tables).

**Table 4:** Thromboprophylaxis practice adequacy.

	Intervention group			Control group			Adjusted difference in change (95% CI), percentage points*†	p value
	Period		Adjusted absolute change, %*§	Period		Adjusted absolute change, %*§		
	Pre-intervention	Intervention		Pre-intervention	Intervention			
	N = 712	N = 8359		N = 690	N = 6992			
Adequate prevention practices—no. (%)	373 (52.4)	4254 (50.9)	-1.4	339 (49.1)	3413 (48.8)	-0.2	-1.2 (-6.6 to 4.2)	0.65
Prophylactic anticoagulation given as recommended in patients with indication—no. (%)	73 (10.3)	1474 (17.6)	5.5	81 (11.7)	1094 (15.6)	3.7	1.7 (-1.8 to 5.2)	0.33
No prophylactic treatment in patients without indication—no. (%)	276 (38.8)	2517 (30.1)	-7.3	238 (34.5)	2060 (29.5)	-4.9	-2.4 (-7.4 to 2.6)	0.34
No prophylactic treatment in patients contraindicated to prophylactic anticoagulation—no. (%)	24 (3.4)	263 (3.1)	-0.2	20 (2.9)	259 (3.7)	0.9	-1.0 (-2.9 to 0.8)	0.25
Inadequate prevention practices—no. (%)	339 (47.6)	4105 (49.1)		351 (50.9)	3579 (51.2)			
Treatment recommended but not given—no. (%)	163 (22.9)	1553 (18.6)	-3.4	151 (21.9)	1587 (22.7)	0.8	-4.3 (-8.9 to 3.9)	0.072
Treatment recommended, but not given as recommended—no. (%)	83 (11.7)	1195 (14.3)	1.8	86 (12.5)	900 (12.9)	-0.1	1.9 (-1.2 to 5.2)	0.24
Treatment not recommended (or contraindicated) but given—no. (%)	93 (13.1)	1357 (16.2)	2.3	114 (16.5)	1092 (15.6)	-0.2	2.5 (-1.1 to 6.1)	0.17

* Adjusted for cluster effect, age, history of chronic respiratory disease, active malignant condition, antiplatelet therapy, surgery during hospitalization, indwelling central venous catheter or cardiac stimulator implantation, length of hospitalization

§ Adjusted absolute change in the frequency of adequate prevention practice between the pre-intervention and intervention periods

† Difference in absolute change of adequacy between the intervention and control groups

CI: confidence interval

Indeed, in the intervention group, the rate of high-risk patients for whom prophylaxis was indicated (Table 1) and who received adequate antithrombotic treatment increased during the intervention period (+6.8%), though this was accompanied by an increase in the rate of high-risk patients who received inadequate treatment because of inadequate dosage, delay, or duration (+2.3%) and in the rate of patients who received treatment when prophylaxis was not indicated or antithrombotic treatment was contraindicated (+3.4%). In the control group, the rate of patients for whom prophylaxis was indicated and who received adequate antithrombotic treatment increased (+5.0%), yet the rate of treatment remained stable for the other patients (Table 4).

The overall proportion of patients who received prophylactic anticoagulant treatment, regardless of the adequacy of the prescription, increased significantly more in the intervention group (+13.2% [35.0% to 48.2%]) than in the control group (+3.4% [40.7% to 44.1%]) (adjusted between-group difference in the change: 6.6% [1.6 to 11.6]; post-hoc analysis; S7 Table).

## Discussion

In this cluster-randomized trial, a multifaceted intervention aimed at implementing venous thromboembolism prophylaxis recommendations did not reduce the rate of symptomatic thromboembolic events or major bleedings within 3 months following hospitalization for acute medical illness. Although it led to an increase in the rate of antithrombotic treatment use, the intervention did not increase the rate of adequate prophylaxis.

Several explanations might account for these negative and unexpected results. First, we planned to implement a multifaceted intervention including a computer-based clinical decision support system and computerized reminders in the intervention group because such interventions appear to be the most effective system-wide measures for improving the quality of care in hospitals.[8, 16] Although all other components of the intervention were implemented in all sites in the intervention group, we were only able to implement plugin reminders in two centers. Two sites had no computerized medical file, four had a computerized file incompatible with the plugin and in five centers, hospital policy did not allow plugin implementation. Moreover, the plugin that we implemented in these two centers could not select high-risk patients and provided reminder for all hospitalized medical patients. This result emphasizes that, complex interventions based on information technology systems and equipment remain difficult to generalize.[17]

Second, the intervention did not increase the rate of adequate prophylaxis. Although an increase in the rate of prophylaxis was observed in the intervention group, this was partially explained by an increase in over-treatment in patients who were not at risk, along with an increase in inappropriate treatment in others. Of note, these outcomes had hardly ever previously been studied. Most previous trials were focused on high-risk patients and did not analyze change *versus* baseline prescription rates.[17–22] Only one previous study demonstrated a reduction in over-treatment using a specific anticoagulant prescription form, [23] yet other reports echo our own in their apparent increases in over-treatment.[24] No prior study has assessed the rate of inadequate treatment, even though inappropriate dosages appear inefficient in the prevention of VTE and over-treatment may expose patients to an unnecessary risk of bleeding.[25]

Third, assessing the thromboembolic risk in medical patients is not always straightforward. Current recommendations are mostly based on the main reason for admission, but a clear definition of most diseases known to be at-risk of VTE is lacking. This is at least the case for acute respiratory failure and rheumatic disorders. The diagnosis may also be uncertain at the time of admission and the definition of bedrest may vary across physicians. A simpler and more reliable risk assessment model may be more effective for a system-wide intervention.[26]

To our knowledge, this study is the first randomized trial planned to assess the clinical benefit-risk ratio of such a multifaceted intervention in unselected hospitalized patients. Among the ten prior randomized studies [17–24, 27], only one demonstrated a decrease in thromboembolic events.[20] This single-center study was based on a complex strategy based on the analysis of the computerized medical files and using electronic alerts in a selected group of untreated high-risk medical and surgical patients (80% patients with underlying cancer). The other studies reported so far were weakened by their study design (primarily before-and-after cycle), size, and outcome criteria (most often not clinical events).[8] A recent meta-analysis of randomized control trials evaluating different interventions to improve thromboprophylaxis has reported an increase in the proportion of high-risk patients receiving treatment, though has failed to demonstrate an improvement in clinical events.[8] Our study, including a large number of patients providing a high power to detect a clinically significant improvement in outcomes, confirms these results.

The main strengths of our study are its size, design, and primary outcome, assessing the clinical benefit of the intervention rather than processes of care. Although the number of participating centers was 27 instead of 30, the statistical power and the cluster-randomized design, allowing us to check for time effect and avoid contamination bias, give us confidence in our results. The study also has some limitations. Firstly, some of our criteria for adequate prophylaxis based on 2008 guidelines may today be debatable.[26, 28] Secondly, we focused our analysis on antithrombotic treatment and did not assess mechanical thromboprophylaxis. Thirdly, due to the lack of stratification by the centers’ activities, there were some differences in the number of patients and patients’ characteristics between the two groups. Fouthly, the rate of thromboembolic events in the control group was lower than expected. Physicians were aware of an ongoing study of thromboprophylaxis and may have improved their baseline practice (Hawthorne effect). Finally, ICU admission and invasive treatments were not recorded.

In conclusion, our multifaceted intervention, aiming to implement current recommendations on thromboprophylaxis for unselected medical patients, failed to demonstrate any clinical benefit. The intervention was associated with an increase in the use of antithrombotic treatment but no increase in adequate thromboprophylaxis was observed. New strategies are required to address thromboprophylaxis in hospitalized medical patients, including simpler assessment of the risk of VTE, at least in hospitals where complex strategies based on computerized reminders and alerts are not implementable.

### Statistical analysis

The statistical analysis was performed by Dr Antoine Rachas and supervised by Pr Gilles Chatellier (Centre d’investigation Épidémiologique 4, INSERM, Paris, France; Unité d’Épidémiologie et de Recherche Clinique, Assistance Publique Hopitaux de Paris, Hôpital Européen Georges Pompidou, Paris, France).

## Supporting Information

S1 CONSORT Checklist CONSORT extension for cluster trial checklist. (DOCX)

S1 Poster Poster A used in the intervention group (in French). (PDF)

S2 Poster Poster B used in the intervention group (in French). (PDF)

S1 Protocol Study protocol (English version). (DOCX)

S1 Statistical Analysis Plan (PDF)

S1 Table Baseline characteristics of patients included during the intervention period by lost to follow-up status. (DOC)

S2 Table Subgroups analysis of clinical events. (DOC)

S3 Table Sensitivity analyses of the main outcome. (DOC)

S4 Table In-hospital outcomes. (DOC)

S5 Table Thromboprophylaxis practices adequacy according to whether a preventive anticoagulant treatment was recommended. (DOC)

S6 Table Thromboprophylaxis practices adequacy and delay of prescription. (DOC)

S7 Table Prescription of prophylactic anticoagulant treatment. (DOC)

S8 Table Three-months outcomes in the 2 hospitals for which a computerized reminder was implemented. (DOC)

S9 Table Thromboprophylaxis practices adequacy in the 2 hospitals for which a computerized reminder was implemented. (DOC)

S10 Table Intracluster correlation coefficients. (DOC)
