# Supplementary material for: Multifaceted Intervention to Prevent Venous Thromboembolism in Patients Hospitalized for Acute Medical Illness: A Multicenter Cluster-Randomized Trial
Source: PLoS One. 2016 May 26;11(5):e0154832. doi: 10.1371/journal.pone.0154832 (PMC4881951; doi:10.1371/journal.pone.0154832)
Supplement: S1 Poster — (PDF) [file pone.0154832.s002.pdf]

Phlébite et Embolie Pulmonaire

Traitement préventif dès les Urgences

Pour qui ?

Les patients avec réduction de mobilité et ayant :

Une situation aiguë à risque important ou majeur

- Insuffisance cardiaque décompensée
- Affection respiratoire aiguë
- Déficit moteur récent touchant un des membres inférieurs (<30 jours)
- Infarctus du myocarde – SCA (<30 jours)

OU

Une situation à risque intermédiaire

- Infection aiguë sévère
- Affection rhumatologique aiguë
- Pathologie inflammatoire intestinale aiguë

ET un ou plusieurs facteurs de risques

- Age supérieur à 75 ans
- Insuffisance cardiaque chronique
- Insuffisance respiratoire chronique
- Cancer évolutif
- Syndrome myéloprolifératif
- Antécédent thrombo-embolique personnel
- Insuffisance veineuse chronique
- Traitement hormonal oestrogénique
- Grossesse et post-partum (<30 jours)
- BMI > 30

Comment ?

Pendant la durée de limitation de mobilité jusqu'à 14 jours.
Se référer aux AMM et recommandations

Principaux traitements possibles :

- **En l'absence d'insuffisance rénale**

Enoxaparine (Lovenox®)
4000 UI anti-Xa/0,4 ml x 1 injection SC / jour

Daltéparine (Fragmine®)
5000 UI anti-Xa/0,2 ml x 1 injection SC / jour

Fondaparinux (Arixtra®)
2,5 mg x 1 injection SC / jour

- **En cas d'insuffisance rénale modérée** **(clairance entre 20 et 50 ml/min)**

Enoxaparine (Lovenox®)
4000 UI anti-Xa/0,4 ml x 1 injection SC / jour

Daltéparine (Fragmine®)
5000 UI anti-Xa/0,2 ml x 1 injection SC / jour

Fondaparinux (Arixtra®)
1,5 mg x 1 injection SC / jour

- **En cas d'insuffisance rénale sévère** **(clairance < 20 ml/min)**

Héparine calcique (Calciparine®)
5000 UI/0,2 ml x 2 (ou 3) injections SC / jour

- **Dans tous les cas**

Contention veineuse stade 2
Contention pneumatique intermittente si CI à un traitement anticoagulant (hémorragie)
