# Supplementary material for: Multifaceted Intervention to Prevent Venous Thromboembolism in Patients Hospitalized for Acute Medical Illness: A Multicenter Cluster-Randomized Trial
Source: PLoS One. 2016 May 26;11(5):e0154832. doi: 10.1371/journal.pone.0154832 (PMC4881951; doi:10.1371/journal.pone.0154832)
Supplement: S2 Poster — (PDF) [file pone.0154832.s003.pdf]

Phlébite et embolie pulmonaire

mieux les connaître pour mieux les éviter

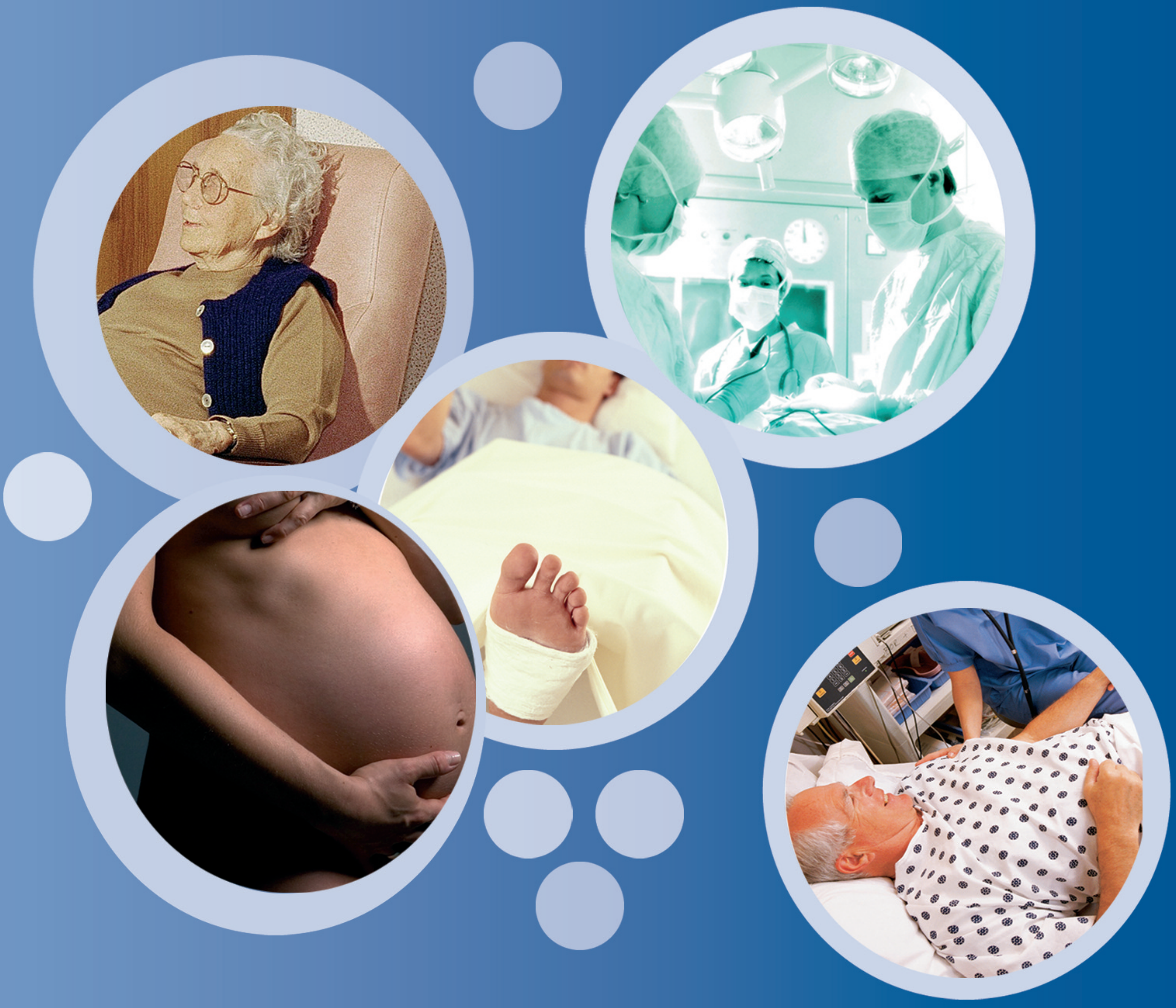

1ère cause de maladie acquise à l'hôpital

La prévention débute dès les Urgences

PREVENU

LIVE

LIgue française contre la maladie VEineuse thrombo-embolique
