# Supplementary material for: Multifaceted Intervention to Prevent Venous Thromboembolism in Patients Hospitalized for Acute Medical Illness: A Multicenter Cluster-Randomized Trial
Source: PLoS One. 2016 May 26;11(5):e0154832. doi: 10.1371/journal.pone.0154832 (PMC4881951; doi:10.1371/journal.pone.0154832)
Supplement: S1 Protocol — (DOCX) [file pone.0154832.s004.docx]

**Prevention of venous thromboembolic disease**

**related to a hospitalisation for Acute medical disorder**

**Interest of a systematic risk assessment and reminder of indications of preventive treatment starting at the emergency department**

*Multicentre cluster randomised impact study*

PREVENU

Prevention of the venous thromboembolic disease starting at the Emergency Department

**Version no. 1 dated 01/07/2008**

**Principal coordinating investigator**

Professor Pierre-Marie Roy, MD, PhD

University and Medical school of Angers

Clinical Research Centre

Trauma and Emergency Department

Centre Hospitalier Universitaire

4 rue Larrey

49933 ANGERS Cedex 9

Telephone: +33 2 41 35 37 18 Fax: +33 2 41 35 40 27

Email: [PMRoy@chu-angers.fr](mailto:PMRoy@chu-angers.fr)

**Sponsor:**

Centre Hospitalier Universitaire d'Angers

4 rue Larrey

499933 ANGERS Cedex 9

**Table of contents**

[1. State of affairs 3](#_Toc436209165)

[1.1 Introduction 3](#_Toc436209166)

[1.2 Venous thromboembolic disease related to hospitalisation in medical setting 3](#_Toc436209167)

[Table 1: Thrombotic risk factors in hospitalised patients in a medical setting. 3](#_Toc436209168)

[1.3 Prevention of VTE in medical settings: from scientific data to daily practice 4](#_Toc436209169)

[1.4 Interventions with the objective of improving prevention practices 5](#_Toc436209170)

[2. Objectives and type of study 5](#_Toc436209171)

[2.1 Main aim 5](#_Toc436209172)

[2.2 Secondary goals 6](#_Toc436209173)

[3. Patients and methods 6](#_Toc436209174)

[3.1 Study population 6](#_Toc436209175)

[3.1.1 Centres 6](#_Toc436209176)

[Table 2: PREVENU study centres 6](#_Toc436209177)

[3.1.2 Patients 7](#_Toc436209178)

[3.1.3 Inclusion process 7](#_Toc436209179)

[3.2 Study scheme and interventions 7](#_Toc436209180)

[3.2.1 Randomisation 7](#_Toc436209181)

[3.2.2 Intervention 8](#_Toc436209182)

[Table 3: Situations at risk of thrombosis that justify initiating a preventive anticoagulant treatment in a hospitalised patient totally or partially confined to bed. 8](#_Toc436209183)

[3.3 Outcome measures 8](#_Toc436209184)

[3.3 Main outcome measure 8](#_Toc436209185)

[3.3 Secondary outcome measures 9](#_Toc436209186)

[3.4 Collection of information 9](#_Toc436209187)

[3.4.1 Evaluation of outcome measures 9](#_Toc436209188)

[3.4.2. Collection support 9](#_Toc436209189)

[4. Statistical analysis 9](#_Toc436209190)

[4.1 Number of subjects required 9](#_Toc436209191)

[4.2 Analysis plan 10](#_Toc436209192)

[5. Discussion 10](#_Toc436209193)

[5.1 Method – type of study 10](#_Toc436209194)

[5.2 Outcome measures and study programme 11](#_Toc436209195)

[6. Ethical and regulatory aspects 11](#_Toc436209196)

[6.1 Compliance with the legislation in force 11](#_Toc436209197)

[6.2 Data Protection 11](#_Toc436209198)

[6.3 Patient information 11](#_Toc436209199)

[6.4 Anonymity of the subjects 12](#_Toc436209200)

[7. Schedule 12](#_Toc436209201)

[8. Literature references 12](#_Toc436209202)

# 1. State of affairs

## 1.1 Introduction

The term venous thromboembolic disease (VTE) includes deep venous thrombosis of the lower limbs (DVT) and pulmonary embolism (PE). VTE is a common disease with annual incidence in the general population estimated at between 1.1 and 1.8 per 1000 persons with a clear increase with age, reaching nearly 1 per 100 per year after the age of 75 years. ^1-4^

It is a serious disease responsible for considerable morbidity-mortality. The incidence of PE has been estimated at approximately 40,000 cases per year in France. ^1-3^

However, considerable progress has been achieved over the last 20 years both on a diagnostic and therapeutic level. When the diagnosis is made, the clinical and paraclinical elements allow estimation of the severity and risk of death and to stratify the therapeutic treatment.^13-16^

In spite all of this, the most striking point of recent epidemiological data is that neither the incidence of VTE, nor PE-related mortality have decreased over the last 20 years. ^5-7, 20, 21^

Therefore, the prevention of VTE in a medical setting seems the principal mean of action for reducing its morbidity-mortality and probably the only way to attain the objectives set by the public health law of 9 August 2004 for the reduction of venous thromboembolic disease by 15% before 2008 (Journal Officiel no. 185 of 11 August 2004 page 14277).

## 1.2 Venous thromboembolic disease related to hospitalisation in medical setting

VTE is a multi-factorial pathology, which involves venous stasis, blood hypercoagulation and vascular wall lesions. It generally appears when there are favouring circumstances (confinement to bed, surgery, immobilisation due to plaster, pregnancy or post-partum, hormone treatment, etc.) in a predisposed patient (age, genetic background, cancer, auto-immunity, etc.).^24^

It has been demonstrated that acute medical conditions have been a major circumstance in the occurrence of VTE. Fifty to 70% of symptomatic thromboembolic accidents arise outside any surgery or trauma, and this is the case for 70 to 80% of fatal PEs.^26^

Out of a population of 1000 patients hospitalised in medicine, 78% have at least one thrombotic risk factor, 48% have two risk factors or more, and 20% three risk factors or more.^27^

##### Table 1: Thrombotic risk factors in hospitalised patients in a medical setting.

| **Risk factor** | **Frequency** |
| --- | --- |
| Chronic respiratory failure | 53.4% |
| Age > 75 years | 50.3% |
| Chronic heart failure | 32% |
| Venous insufficiency | 25% |
| Obesity | 20% |
| Progressive cancer | 14% |
| Personal thromboembolic history | 9.4% |
| Hormone treatment | 2% |

From Samama et al., N Engl J Med, 1999^28^

15 to 17% of patients hospitalised in medicine departments develop venous thromboembolic disease during their stay: 1% pulmonary embolism, 4% symptomatic DVT and 10% asymptomatic DVT. ^22, 29, 30^ In the absence of preventive drug therapy, the prevalence of thromboembolic accidents in patients hospitalised for stroke is estimated at 56% (confidence interval at 95%: 51-61%), while that of patients suffering from a myocardial infarction is at 22% (CI at 95% 16-28%). ^31^ Many thromboembolic accidents occur quite rapidly after the start of the acute disorder. In the work by Oger *et al.* in Brest, 5.5% of patients had a thrombosis from their admission in an internal medicine department (CI at 95%: 3.1 to 9.5%). ^34^ The incidence during hospitalisation was of 2.6 per 1000 days-patient. The prevalence on admission and the incidence significantly increased as a function of age, reaching 17.8% and 6 per 1000 days-patient after 80 years.^34^

The thromboembolic strokes diagnosed during hospital stays only represent a portion of the VTE related to healthcare, as the thrombotic accidents that manifest themselves after hospitalisation are probably the most common. In a study not yet published, the team from Brest demonstrated that 40% of the cases of VTE diagnosed in Finistère occurred in patients who had been hospitalised over the previous three months (more than 80% of these patients had been hospitalised in the medicine department).

## 1.3 Prevention of VTE in medical settings: from scientific data to daily practice

This frequency of thromboembolic strokes related to hospitalisations clashes with the demonstrated efficacy of thromboprophylaxis.^37^ In fact, several studies have demonstrated that in target populations, an antithrombotic treatment significantly reduced thromboses during hospitalisation for an acute medical disorder (annex 1). The three main studies are the MEDENOX study with enoxaparin published in 1999, the PREVENT study with dalteparin published in 2004 and the ARTERMIS study with fondaparinux published in 2006. ^28, 30, 38^ The main criticism made about these research works was their primary outcome measure: thromboembolic strokes detected by a systematic iconographic analysis. Meta-analyses combining their results demonstrated not only the efficacy of drug thromboprophylaxis in medical patients in terms of the clinical events prevented but also its cost-efficacy. ^19, 39, 40^ A prophylactic treatment significantly reduces symptomatic PE (relative risk 0.43 [CI at 95%: 0.26-0.71]), and fatal PE (relative risk 0.38 [CI at 95%: 0.21-0.69]) with a non-significant increase in serious haemorrhagic accidents (relative risk 1.32 [CI at 95%: 0.73-2.37.).^41^ The number of patients treated to prevent a fatal PE has been estimated at 400.^41^

In patients treated at a prophylactic dose in controlled trials, the incidence of the VTE remains between 2.8 and 5.6%. ^29, 30, 38^ This would suggest that certain high risk patients would benefit from specific therapeutic measures such as the combination of venous contention and drug therapy or the choice of a specific treatment or a higher dose. Anti-thrombotic treatments also comprise an iatrogenic risk, in particular that of a serious haemorrhagic stroke.

Above all the impact of the prophylaxis is low because it is hardly and/or poorly applied in everyday practice. The IMPROVE study analysed the VTE prophylaxis practices between July 2002 and September 2006 in 15156 patients hospitalised for medical reasons in 52 hospitals in 12 countries, including France. Only 60% of patients for whom a prophylactic treatment was justifiable according to the ACCP 2004 criteria (annex 2) actually benefited from a preventive measure, including venous contention. In a study carried out in 30 Internal Medicine departments of the AP-HP (Paris area hospitals), in the absence of intervention, 40% of patients with a high thrombotic risk did not receive any prophylactic treatment (under prescription) while 25% of the low risk patients received one (over prescription).^45^

For all these reasons, the prevention of venous thromboembolic disease has been rated at the forefront of interventions to be implemented in hospitals to improve the safety of patients by the American agency for healthcare research and quality (<http://www.ahrq.gov/clinic/ptsafety>, accessed 1 December 2007).

## 1.4 Interventions with the objective of improving prevention practices

In general, recommendations are more likely to be applied if they are appropriate to everyday real practice, if their pertinence is recognised by local decision-makers and that the latter are involved in disseminating the information.^48, 49^

The intervention most commonly undertaken is the preparation of an operational protocol widely distributed in writing. However, its effect is often moderate and transient.^50^ Recommendations based on a computer-support are the most effective probably because they allow a contextual automatic reminder and that the information could be “pushed” towards the clinician without having to go and find it.^51^ Recently, Fontaine *et al.* demonstrated that the set-up of a contextual reminder in the internal medicine departments of the AP-HP integrated to the anti-thrombotic agents prescription paper support resulted in a considerable decrease in over-prescription but had no impact on under-prescription.^45^ Conversely, Durieux *et al.* demonstrated in 2000 the efficacy of a computerised reminder which brought the prescriptions in line with the prophylaxis recommendations in 95% of the cases of patients treated in orthopaedic surgery at a Parisian hospital.^50^ The automatic reminder integrated to a computerised prescription system had a partial efficacy compared to a control group, but significant both in terms of the prophylactic treatment actually received by the patients at risk (34% vs. 15%, P<0.001) and in terms of clinical events at 3 months (4.9% vs. 8.2%, P<0.001).^53^ Conversely, some recent studies have shown the practical difficulties in the use of routine computerised support.^54, 55^ The use of information technology is actually limited by the need to have computerised patient records which only concerns a minority of French hospital centres to date, by the possibilities to integrate the contextual reminder in the software used, by the comprehensiveness of the data on which the reminder will based itself, by the accessibility to a computer and by the time available for the doctors.

In order to have a noticeable impact, the intervention must be able to be adapted to the reality of each centre, especially with respect to the information technology tool and allow improving prevention practices irrespective of the department in which the patient is hospitalised. An intervention in the emergency departments could satisfy these objectives. In fact, the prescriptions made by the emergency unit doctor at the time hospitalisation is decided are followed in the departments if they agree with good practice recommendations.

# 2. Objectives and type of study

## 2.1 Main aim

*Main aim*: Evaluate the efficacy of a systematic assessment of the risk and contextual reminder of anticoagulant treatment recommendations to prevent venous thromboembolic disease in patients hospitalised for an acute medical condition by the Emergency departments.

The primary outcome criterion will be the number of clinical thromboembolic events and serious haemorrhagic strokes diagnosed over the 3 months (90 days) following admission.

## 2.2 Secondary goals

*Secondary goals:*

1) Evaluate the efficacy of the contextual reminder on the quality of medical practice from the proportion of patients who received a treatment in compliance with the recommendations.

2) Evaluate the influence of the method of implementation of the recommendations (paper support or integration into computer software) on the quality of the medical practice.

3) Evaluate the incidence and risk factors of VTE associated with hospitalisation in common medical practice in France from the rate of thromboembolic strokes in the control group.

4) Evaluate the incidence and risk factors of VTE related to insufficient efficacy of current prophylactic treatments from the rate of thromboembolic strokes in patients who have had a preventive treatment in compliance with recommendations.

5) Evaluate the frequency of iatrogenic accidents and incidents secondary to anticoagulant treatments given for the prevention of venous thromboembolic disease.

# 3. Patients and methods

## 3.1 Study population

### 3.1.1 Centres

Thirty emergency departments will participate in this study.

Thirty centres consented to participate in the study are mentioned in table 2. Four other are likely to participate in case of withdrawal.

##### Table 2: PREVENU study centres

| CENTRES | Type | No. of admissions per year |  | INVESTIGATORS and/or HEAD of DEPARTMENT |
| --- | --- | --- | --- | --- |
| Angers | CHU | 45000 |  | Pr Pierre-Marie Roy |
| Argenteuil | CHG | 64000 |  | Dr Catherine Legall |
| Besançon | CHU | 42000 |  | Dr Mohamed Hachelaf |
| Béthune | CHG | 31000 |  | Dr Alain-Eric Dubart |
| Boulogne | CHG | 32000 |  | Pr David Elkharrat |
| Brest | CHU | 41000 |  | Dr Grégoire Legal |
| Caen | CHU | 58000 |  | Dr Aurore Armand-Perroux / Pr Eric Roupie |
| Carcassonne | CHG | 30000 |  | Dr Frédéric Joye |
| Chateauroux | CHG | 33000 |  | Dr Guillem Bouilleau / Dr Louis Soulat |
| Clermont-Ferrand | CHU | 42000 |  | Pr Jeannot Schmidt |
| Compiègne | CHG | 46000 |  | Dr Patrick Miroux |
| Créteil | CHU | 44000 |  | Dr Bertrand Renaud |
| Dijon | CHU | 39000 |  | Dr Didier Honnart |
| Grenoble | CHU | 51000 |  | Pr Françoise Carpentier |
| Le Mans | CHG | 54000 |  | Dr Jacques Choukroun |
| Lille | CHU | 55000 |  | Pr Patrick Goldstein |
| Marseille Hôpital de la Conception | CHU | 47000 |  | Pr Patrick Gerbeaux |
| Mulhouse | CHG | 43000 |  | Dr Jean Rottner |
| Nice | CHU | NC |  | Pr Jacques Levraut |
| Nimes | CHU | 56000 |  | Pr Jean-Emmanuel De la Coussaye |
| Nantes | CHU | 65000 |  | Pr Philippe Leconte / Pr Gilles Potel |
| Paris Pitié-Salpêtrière | CHU | 52000 |  | Dr Pierre Hausfater / Pr Bruno Riou |
| Paris Bobigny | CHU | 36000 |  | Pr Frédéric Lapostolle |
| Paris Hôtel Dieu | CHU | 41000 |  | Pr Jean Louis Pourriat |
| Paris Cochin | CHU | 48000 |  | Dr Yann-Erick Claessens / Pr Jean Louis Pourriat |
| Paris Tenon | CHU | 45000 |  | Dr Etienne Hinglais |
| Poitiers | CHU | 43000 |  | Dr Fatima Rayef |
| Saint Pierre la Réunion | CHG | 37500 |  | Dr Frédérick Staikowsky |
| Strasbourg | CHU | 49000 |  | Dr Laurent Calvel / Pr Jacques Kopferschmidt |

NC: not communicated

### 3.1.2 Patients

*Inclusion criteria*

- age ≥ 40 years

- admission to emergency departments for a non-trauma reason

- hospitalisation in a medicine department from a centre participating in the study

*Non-inclusion criteria*

- age < 40 years

- hospitalisation in another healthcare structure other than the centres participating in the study

- patient receiving an anticoagulant treatment at curative doses on admission

- patient for whom the follow-up at three months would be impossible (end of life, homeless, not reachable)

- patient who refuses to be contacted by telephone 3 months after admission and/or that the information concerning him/her be used for scientific purposes.

*Exclusion criteria*

- patient for whom a diagnosis of venous thromboembolic disease is made within 48 hours following admission

- patient receiving an anticoagulant treatment at a curative dose for more than 5 days for 3 months following admission for another cause other than venous thromboembolic disease.

### 3.1.3 Inclusion process

The daily admissions and hospitalisations daily legal record of the emergency departments will provide an exhaustive list of patients that may be included. A clinical research assistant will ask the patient for an oral consent for the use of his/her data in thePREVENU study and to be contacted by telephone 3 months after the admission.

## 3.2 Study scheme and interventions

### 3.2.1 Randomisation

The PREVENU study will be organised in two phases, a simple assessment of professional practices neutral phase and an active phase corresponding to the assessment of the intervention carried out.

After the neutral simple assessment of practices periods, the participating centres will be randomised in two groups of 15: one control group without intervention on the practices and an intervention group.

The randomisation will be carried out with stratification depending on the possibility or impossibility of integrating a contextual reminder in the information technology tool of the emergency department and, if possible, as a function of the adequacy rate of the practices observed during the first phase of the study.

### 3.2.2 Intervention

In the control group no intervention will be carried out to modify their practices.

In the intervention group, a systematic assessment process for the at risk situation and thrombotic risk factors with reminder of the preventive treatment indications to be implemented.

In as much as possible, it will be integrated to the information technology support of the participating centres.

If no computerised reminder is possible, the reminder will be carried out in paper format only.

In all cases, posters mentioning the same criteria will be provided for the emergency departments.

##### Table 3: Situations at risk of thrombosis that justify initiating a preventive anticoagulant treatment in a hospitalised patient totally or partially confined to bed.

| **Acute situation with considerable or major risk** | |
| --- | --- |
| Decompensated cardiac failure (stage III or IV) | |
| Serious acute respiratory disorder | |
| Recent motor deficit of the lower limbs (< 30 days) | |
| Myocardial infarction of less than 30 days (acute coronary syndrome) | |
| **Acute at risk situation and** | **1 or several Risk factor(s)** |
| Severe acute infection | Age ≥ 75 years |
| Acute rheumatoid disorder | History of thromboembolic disease |
| Acute inflammatory pathology | Progressive cancer |
|  | Obesity (BMI > 30) |
|  | Chronic venous insufficiency |
|  | Oestrogenic hormone treatment |
|  | Chronic heart failure |
|  | Chronic respiratory failure |
|  | Pregnancy and post-partum (< 30 days) |

Treatment is recommended in hospitalised patients when there is an acute situation with a considerable or major risk or when there is an acute situation with intermediate risk with at least one thrombotic risk factor.

## 3.3 Outcome measures

### 3.3 Main outcome measure

The main outcome measure will be the rate of symptomatic venous thromboembolic events and/or serious haemorrhagic strokes diagnosed within 3 months following the admission (up to the 90^th^ day inclusive) excluding the first two days.

In the main analysis only the first event will be taken into account for the patients who suffer several thromboembolic or haemorrhagic events.

### 3.3 Secondary outcome measures

The quality of venous thromboembolic disease prevention practices

- - The first outcome measure for the analysis of the practice is the evolution of the proportion of patients who receive a treatment in accordance with the recommendations between the first and second period of the study (pre- and post-intervention study).
  - Poor practice criteria analysed individually

Preventive treatment benefit/risk ratio

- Efficacy analysis criteria
  - venous thrombosis rate
  - pulmonary embolism rate
  - fatal pulmonary embolism rate
  - all thromboembolic events rate

- The preventive treatment safety criteria

- - serious haemorrhagic stroke rate
  - non-serious haemorrhagic stroke rate
  - heparin-induced thrombocytopoenia rate
- Mortality at 3-months including all causes

## 3.4 Collection of information

### 3.4.1 Evaluation of outcome measures

The thromboembolic events and haemorrhagic strokes diagnosed during hospitalisation will be considered from the hospitalisation reports.

The suspected venous thromboembolic events or haemorrhagic strokes occurring within 3 months will be examined in detail to look for results of examinations that allow the identification of the thrombosis or haemorrhage. The records of the patients hospitalised within 3 months after inclusion will be closely examined for the causes of the hospitalisation. The introduction of an anticoagulant treatment during the follow-up will be examined, as well as its indication if applicable. The causes of death will be analysed.

### 3.4.2. Collection support

The study data will be collected in an electronic research form which will be developed specifically for the study.

# 4. Statistical analysis

## 4.1 Number of subjects required

Cluster randomisation trials use specific rules for the estimate of the number of subjects, taking into account the centre effect responsible of a non-independence of the patients from a the same centre characterised by an intra-class coefficient of correlation.

The incidence of clinical thromboembolic events and haemorrhagic strokes in the placebo group of therapeutic studies and in control groups of intervention studies ranges between 1% and 10% with variations depending on the duration of the follow-up, the definition of the outcome measurements and the typology of the patients.^28, 30, 53, 88-93^

We estimated that it would be of 5% at 3 months in the control group and that the intervention would reduce the thromboembolic strokes without increasing the haemorrhagic strokes with an absolute difference on the main outcome measure of 1.5% i.e. a serious haemorrhagic stroke or thrombotic event rate of 3.5% in the intervention group.

Considering the participation of 30 centres, 15 in each group, with a rate of expected events in the control group of 5% and of 3.5% in the intervention group, an intra-class coefficient of correlation of 0.01, 16170 patients would be required to demonstrate a significant difference with a 5% alpha risk and a 20% beta risk. Considering the exclusions and the possibility of patients lost at follow-up, a population of 16500 was taken into account, i.e. 550 patients per centre.

This population would also allow response to the secondary objectives.

For the evolution of the adequacy of preventive therapeutic practices, considering an adequacy rate of 50% in the control group and 70% in the intervention group, and an intra-class coefficient of correlation of 0.25, 1294 patients would be required to demonstrate a significant difference with a 1% alpha risk and a 10% beta risk.

Considering that the proportion of patients suffering from symptomatic venous thromboembolic disease or death due to pulmonary embolism within the 3 months following a hospitalisation would be of 4% in the control group, 8100 patients would allow observing 324 thrombotic events. The comparison of 324 cases and 7776 “control” patients allows detecting an odds-ratio greater than or equal to 2 for exposures with a frequency greater than or equal to 10% with a type I error of 5% and a power of 90%.

## 4.2 Analysis plan

Standard statistical methods (t test or chi2 test depending on the case) will be used for the baseline description of the departments and the patients. The statistical analysis will use standard percentage comparison methods. The confidence intervals will be calculated using parametric or non-parametric methods, depending on the validity of the parametric model. The analysis will take into account the cluster effect, i.e. the non-independence of patient observations of each participating centre, with a mixed effect model in which the hospital will be modelled as a nested random effect in the "active" group and the intervention as a fixed effect.^87, 94-96^

# 5. Discussion

## 5.1 Method – type of study

Randomised controlled studies are considered to be the most pertinent, however they require a special analysis.^94^ In these studies, the randomisation is carried out in the centres, however the efficacy is measured at the level of the patients which increases the number of subjects to be included to demonstrate an effect and conditions the type of statistical tests to be used.^87, 95, 96^ It is recommended to combine an evaluation of the pre-interventional practices in order to have an accurate idea of the baseline condition and analyse the evolution of the practices via the difference in the adequacy before and after the intervention in the two groups.^52^

The main outcome measure here is a result criterion since if the efficacy of the preventive treatment is demonstrated, its impact in terms of clinical events remains imprecise, as the incidence of the VTE-related to hospitalisation itself is not perfectly defined as well as the risks, in particular haemorrhagic, related to any “abusive” prescription of anti-thrombotic treatments.

## 5.2 Outcome measures and study programme

The choice of a composite criterion grouping as failures of prevention (thromboembolic strokes) and iatrogenic accidents related to it (haemorrhagic strokes) allows a proper assessment of the benefit/risk ratio of the intervention. The definition of thromboembolic events and serious haemorrhagic strokes corresponds to international standards. ^41, 72, 85, 98^

The collection of symptomatic clinical elements will be based on the patient records and a telephone interrogation at 3 months.

# 6. Ethical and regulatory aspects

## 6.1 Compliance with the legislation in force

The investigator agrees to perform the in compliance with the legislation in force: amended law no. 88-1138 of 20 December 1988 (referred to as Huriet-Sérusclat law), law no. 2004-806 of 09/08/2004 concerning public health policy, European Directive 2001/20/CE, decree no. 2006-477 of 26 April 2006 amending chapter I of title II of the first section of the public health code concerning biomedical research (JO of 27 April 2006), Guideline for Good Clinical Practice of the ICH 1996 and the declaration of Helsinki (Edinburgh, 2000).

## 6.2 Data Protection

The information collected will be processed confidentially in accordance with law no. 78-17 concerning information technology, data processing and civil liberties,

## 6.3 Patient information

The PREVENU study is a randomised study but without direct intervention in the care given to the patients. The doctors will be free to follow or not follow the recommendations they will be given. Studies of this type carried out within the framework of the national PHRC have been considered to be outside the framework of the law of 09/08/2004 concerning public health policy by the Clinical Research Department concerned and do not require the informed and signed consent of the patients included. ^48, 50, 84^ However, the patients will be informed of the study and they will be allowed to oppose it.

A poster giving information on the study and the rights of patients concerning their access to their data in accordance with the law in force of 6 January 1978 will be made available for display in the waiting room of the Emergency Admission Department of each investigator centre.

An information note will be given to the patient indicating that the data collected will be processed and that he/she has a right of access and correction of the information concerning him/her at all times.

## 6.4 Anonymity of the subjects

The anonymity of the patients participating in the study will be respected: only the first letter of the surname and the first letter of the first name will be indicated in the electronic case report form and the other study documents.

# 7. Schedule

Considering the activity of the centres likely to participate in the PREVENU study, the inclusion period should last approximately 2 months. Taking into account the initial period for the assessment of practices prior to intervention of one week and the follow-up period of 3 months, the active period of the study will last approximately 6 months.

The organisation of the PREVENU study should start in September 2009. The initial practices evaluation phase of one week should be carried out at the end of September. The active phase should start on the first of October 2009 for an end of inclusions in December and an end of the follow-up period at the end of March 2010.

# 8. Literature references

1. Oger E. Incidence of venous thromboembolism: a community-based study in Western France. EPI-GETBO Study Group. Groupe d'Etude de la Thrombose de Bretagne Occidentale. Thromb Haemost 2000;83(5):657-60.

2. Heit JA, Silverstein MD, Mohr DN, Petterson TM, O'Fallon WM, Melton LJ, 3rd. Risk factors for deep vein thrombosis and pulmonary embolism: a population-based case-control study. Arch Intern Med 2000;160(6):809-15.

3. Fowkes FJ, Price JF, Fowkes FG. Incidence of diagnosed deep vein thrombosis in the general population: systematic review. Eur J Vasc Endovasc Surg 2003;25(1):1-5.

4. Stein PD, Hull RD, Kayali F, Ghali WA, Alshab AK, Olson RE. Venous thromboembolism according to age. The impact of an aging population. Arch Intern Med 2004;164:2260-5.

5. Ferrari E, Baudouy M, Cerboni P, et al. Clinical epidemiology of venous thromboembolic disease. Results of a French Multicentre Registry. Eur Heart J 1997;18(4):685-91.

6. Goldhaber SZ, Visani L, De Rosa M. Acute pulmonary embolism: clinical outcomes in the International Cooperative Pulmonary Embolism Registry (ICOPER). Lancet 1999;353(9162):1386-9.

7. Murin S, Romano PS, White RH. Comparison of outcomes after hospitalization for deep venous thrombosis or pulmonary embolism. Thromb Haemost 2002;88(3):407-14.

13. Aujesky D, Roy PM, Le Manach CP, et al. Validation of a model to predict adverse outcomes in patients with pulmonary embolism. Eur Heart J 2006;27(4):476-81.

14. Wicki J, Perrier A, Perneger T, Bounameaux H, Junod A. Predicting adverse outcome in patients with acute pulmonary embolism: a risk score. Thromb Haemost 2000;84:548-52.

15. Scridon T, Scridon C, Skali H, Alvarez A, Goldhaber SZ, Solomon SD. Prognostic significance of troponin elevation and right ventricular enlargement in acute pulmonary embolism. Am J Cardiol 2005;96(2):303-5.

16. Kucher N, Rossi E, De Rosa M, Goldhaber SZ. Prognostic role of echocardiography among patients with acute pulmonary embolism and a systolic arterial pressure of 90 mm Hg or higher. Arch Intern Med 2005;165(15):1777-81.

19. Buller HR, Agnelli G, Hull RD, Hyers TM, Prins MH, Raskob GE. Antithrombotic therapy for venous thromboembolic disease: the Seventh ACCP Conference on Antithrombotic and Thrombolytic Therapy. Chest 2004;126(3 Suppl):401S-28S.

20. Morpurgo M, Schmid C, Mandelli V. Factors influencing the clinical diagnosis of pulmonary embolism: analysis of 229 postmortem cases. Int J Cardiol 1998;65 Suppl 1:S79-82.

21. Stein PD, Kayali F, Olson RE. Estimated case fatality rate of pulmonary embolism, 1979 to 1998. Am J Cardiol 2004;93(9):1197-9.

22. Stein PD, Henry JW. Prevalence of acute pulmonary embolism among patients in a general hospital and at autopsy. Chest 1995;108(4):978-81.

24. Rosendaal F. Venous thrombosis: a multicausal disease. The Lancet 1999;353(9159):1167-73.

26. Goldhaber SZ. Thromboembolism management in Europe vs North America: are the differences clinically significant? Chest 2000;117(6):1536-7.

27. Anderson FA, Jr, Wheeler HB, Goldberg RJ, Hosmer DW, Forcier A. The prevalence of risk factors for venous thromboembolism among hospital patients

10.1001/archinte.152.8.1660. Arch Intern Med 1992;152(8):1660-4.

28. Samama MM, Cohen AT, Darmon JY, et al. A comparison of enoxaparin with placebo for the prevention of venous thromboembolism in acutely ill medical patients. Prophylaxis in Medical Patients with Enoxaparin Study Group. N Engl J Med 1999;341(11):793-800.

29. Samama MM, Cohen AT, Darmon JY, et al. A comparison of enoxaparin with placebo for the prevention of venous thromboembolism in acutely ill medical patients. N Engl J Med 1999;341:793-800.

30. Leizorovicz A, Cohen AT, Turpie AG, Olsson CG, Vaitkus PT, Goldhaber SZ. Randomized, placebo-controlled trial of dalteparin for the prevention of venous thromboembolism in acutely ill medical patients. Circulation 2004;110(7):874-9.

31. Cohen AT, Alikhan R, Arcelus JI, et al. Assessment of venous thromboembolism risk and the benefits of thromboprophylaxis in medical patients. Thromb Haemost 2005;94(4):750-9.

34. Oger E, Bressollette L, Nonent M, et al. High prevalence of asymptomatic deep vein thrombosis on admission in a medical unit among elderly patients. The TADEUS Project. Thromb Haemost 2002;88:592-7.

37. Geerts WH, Pineo GF, Heit JA, et al. Prevention of venous thromboembolism: the Seventh ACCP Conference on Antithrombotic and Thrombolytic Therapy. Chest 2004;126(3 Suppl):338S-400S.

38. Cohen AT, Davidson BL, Gallus AS, et al. Efficacy and safety of fondaparinux for the prevention of venous thromboembolism in older acute medical patients: randomised placebo controlled trial. Bmj 2006;332(7537):325-9.

39. de Lissovoy G, Subedi P. Economic evaluation of enoxaparin as prophylaxis against venous thromboembolism in seriously ill medical patients: a US perspective. Am J Manag Care 2002;8(12):1082-8.

40. Wade WE, Chisholm MA. Cost-effectiveness analysis of deep vein thrombosis prophylaxis in internal medicine patients. Thromb Res 1999;94(1):65-8.

41. Dentali F, Douketis JD, Gianni M, Lim W, Crowther MA. Meta-analysis: anticoagulant prophylaxis to prevent symptomatic venous thromboembolism in hospitalized medical patients. Ann Intern Med 2007;146(4):278-88.

45. Fontaine A, Mahe I, Bergmann JF, et al. Effectiveness of written guidelines on the appropriateness of thromboprophylaxis prescriptions for medical patients: a prospective randomized study. J Intern Med 2006;260(4):369-76.

48. Durieux P, Ravaud P, Dosquet P, Durocher A. [Effectiveness of clinical guideline implementation strategies: systematic review of systematic reviews]. Gastroenterol Clin Biol 2000;24(11):1018-25.

49. Thomson O'Brien MA, Oxman AD, Haynes RB, Davis DA, Freementle N, Harvey EL. Local opinion leaders: effects on professional practice and health care outcomes (Cochrane reviews). In: The Cochrane Library. Oxford: Update Software; 2001.

50. Durieux P, Nizard R, Ravaud P, Mounier N, Lepage E. A clinical decision support system for prevention of venous thromboembolism: effect on physician behavior. Jama 2000;283(21):2816-21.

51. Tooher R, Middleton P, Pham C, et al. A systematic review of strategies to improve prophylaxis for venous thromboembolism in hospitals. Annals of surgery 2005;241(3):397-415.

52. Grimshaw J, Campbell M, Eccles M, Steen N. Experimental and quasi-experimental designs for evaluating guideline implementation strategies. Fam Pract 2000;17 Suppl 1:S11-6.

53. Kucher N, Koo S, Quiroz R, et al. Electronic alerts to prevent venous thromboembolism among hospitalized patients. N Engl J Med 2005;352(10):969-77.

72. Perrier A, Roy PM, Sanchez O, et al. Multidetector-row computed tomography in suspected pulmonary embolism. N Engl J Med 2005;352(17):1760-8.

84. Auleley GR, Ravaud P, Giraudeau B, et al. Implementation of the Ottawa ankle rules in France. A multicenter randomized controlled trial. Jama 1997;277(24):1935-9.

85. Schulman S, Kearon C. Definition of major bleeding in clinical investigations of antihemostatic medicinal products in non-surgical patients. J Thromb Haemost 2005;3(4):692-4.

87. Donner A, Klar N. Statistical considerations in the design and analysis of community intervention trials. J Clin Epidemiol 1996;49(4):435-9.

88. Belch JJ, Lowe GD, Ward AG, Forbes CD, Prentice CR. Prevention of deep vein thrombosis in medical patients by low-dose heparin. Scott Med J 1981;26(2):115-7.

89. Dahan R, Houlbert D, Caulin C, et al. Prevention of deep vein thrombosis in elderly medical in-patients by a low molecular weight heparin: a randomized double-blind trial. Haemostasis 1986;16(2):159-64.

90. Gardlund B. Randomised, controlled trial of low-dose heparin for prevention of fatal pulmonary embolism in patients with infectious diseases. The Heparin Prophylaxis Study Group. Lancet 1996;347(9012):1357-61.

91. McGarry LJ, Thompson D. Retrospective database analysis of the prevention of venous thromboembolism with low-molecular-weight heparin in acutely III medical inpatients in community practice. Clinical therapeutics 2004;26(3):419-30.

92. Lederle FA, Sacks JM, Fiore L, et al. The prophylaxis of medical patients for thromboembolism pilot study. Am J Med 2006;119(1):54-9.

93. Leizorovicz A, Mismetti P. Preventing venous thromboembolism in medical patients. Circulation 2004;110(24 Suppl 1):IV13-9.

94. Klar N, Donner A. Current and future challenges in the design and analysis of cluster randomization trials. Stat Med 2001;20(24):3729-40.

95. Kerry SM, Bland JM. The intracluster correlation coefficient in cluster randomisation. Bmj 1998;316(7142):1455.

96. Kerry SM, Bland JM. Sample size in cluster randomisation. Bmj 1998;316(7130):549.

98. Stein PD, Fowler SE, Goodman LR, et al. Multidetector computed tomography for acute pulmonary embolism. N Engl J Med 2006;354(22):2317-27.

1. Oger E. Incidence of venous thromboembolism: a community-based study in Western France. EPI-GETBO Study Group. Groupe d'Etude de la Thrombose de Bretagne Occidentale. Thromb Haemost 2000;83(5):657-60.

2. Heit JA, Silverstein MD, Mohr DN, Petterson TM, O'Fallon WM, Melton LJ, 3rd. Risk factors for deep vein thrombosis and pulmonary embolism: a population-based case-control study. Arch Intern Med 2000;160(6):809-15.

3. Fowkes FJ, Price JF, Fowkes FG. Incidence of diagnosed deep vein thrombosis in the general population: systematic review. Eur J Vasc Endovasc Surg 2003;25(1):1-5.

4. Stein PD, Hull RD, Kayali F, Ghali WA, Alshab AK, Olson RE. Venous thromboembolism according to age. The impact of an aging population. Arch Intern Med 2004;164:2260-5.

5. Ferrari E, Baudouy M, Cerboni P, et al. Clinical epidemiology of venous thromboembolic disease. Results of a French Multicentre Registry. Eur Heart J 1997;18(4):685-91.

6. Goldhaber SZ, Visani L, De Rosa M. Acute pulmonary embolism: clinical outcomes in the International Cooperative Pulmonary Embolism Registry (ICOPER). Lancet 1999;353(9162):1386-9.

7. Murin S, Romano PS, White RH. Comparison of outcomes after hospitalization for deep venous thrombosis or pulmonary embolism. Thromb Haemost 2002;88(3):407-14.

13. Aujesky D, Roy PM, Le Manach CP, et al. Validation of a model to predict adverse outcomes in patients with pulmonary embolism. Eur Heart J 2006;27(4):476-81.

14. Wicki J, Perrier A, Perneger T, Bounameaux H, Junod A. Predicting adverse outcome in patients with acute pulmonary embolism: a risk score. Thromb Haemost 2000;84:548-52.

15. Scridon T, Scridon C, Skali H, Alvarez A, Goldhaber SZ, Solomon SD. Prognostic significance of troponin elevation and right ventricular enlargement in acute pulmonary embolism. Am J Cardiol 2005;96(2):303-5.

16. Kucher N, Rossi E, De Rosa M, Goldhaber SZ. Prognostic role of echocardiography among patients with acute pulmonary embolism and a systolic arterial pressure of 90 mm Hg or higher. Arch Intern Med 2005;165(15):1777-81.

19. Buller HR, Agnelli G, Hull RD, Hyers TM, Prins MH, Raskob GE. Antithrombotic therapy for venous thromboembolic disease: the Seventh ACCP Conference on Antithrombotic and Thrombolytic Therapy. Chest 2004;126(3 Suppl):401S-28S.

20. Morpurgo M, Schmid C, Mandelli V. Factors influencing the clinical diagnosis of pulmonary embolism: analysis of 229 postmortem cases. Int J Cardiol 1998;65 Suppl 1:S79-82.

21. Stein PD, Kayali F, Olson RE. Estimated case fatality rate of pulmonary embolism, 1979 to 1998. Am J Cardiol 2004;93(9):1197-9.

22. Stein PD, Henry JW. Prevalence of acute pulmonary embolism among patients in a general hospital and at autopsy. Chest 1995;108(4):978-81.

24. Rosendaal F. Venous thrombosis: a multicausal disease. The Lancet 1999;353(9159):1167-73.

26. Goldhaber SZ. Thromboembolism management in Europe vs North America: are the differences clinically significant? Chest 2000;117(6):1536-7.

27. Anderson FA, Jr, Wheeler HB, Goldberg RJ, Hosmer DW, Forcier A. The prevalence of risk factors for venous thromboembolism among hospital patients

10.1001/archinte.152.8.1660. Arch Intern Med 1992;152(8):1660-4.

28. Samama MM, Cohen AT, Darmon JY, et al. A comparison of enoxaparin with placebo for the prevention of venous thromboembolism in acutely ill medical patients. Prophylaxis in Medical Patients with Enoxaparin Study Group. N Engl J Med 1999;341(11):793-800.

29. Samama MM, Cohen AT, Darmon JY, et al. A comparison of enoxaparin with placebo for the prevention of venous thromboembolism in acutely ill medical patients. N Engl J Med 1999;341:793-800.

30. Leizorovicz A, Cohen AT, Turpie AG, Olsson CG, Vaitkus PT, Goldhaber SZ. Randomized, placebo-controlled trial of dalteparin for the prevention of venous thromboembolism in acutely ill medical patients. Circulation 2004;110(7):874-9.

31. Cohen AT, Alikhan R, Arcelus JI, et al. Assessment of venous thromboembolism risk and the benefits of thromboprophylaxis in medical patients. Thromb Haemost 2005;94(4):750-9.

34. Oger E, Bressollette L, Nonent M, et al. High prevalence of asymptomatic deep vein thrombosis on admission in a medical unit among elderly patients. The TADEUS Project. Thromb Haemost 2002;88:592-7.

37. Geerts WH, Pineo GF, Heit JA, et al. Prevention of venous thromboembolism: the Seventh ACCP Conference on Antithrombotic and Thrombolytic Therapy. Chest 2004;126(3 Suppl):338S-400S.

38. Cohen AT, Davidson BL, Gallus AS, et al. Efficacy and safety of fondaparinux for the prevention of venous thromboembolism in older acute medical patients: randomised placebo controlled trial. Bmj 2006;332(7537):325-9.

39. de Lissovoy G, Subedi P. Economic evaluation of enoxaparin as prophylaxis against venous thromboembolism in seriously ill medical patients: a US perspective. Am J Manag Care 2002;8(12):1082-8.

40. Wade WE, Chisholm MA. Cost-effectiveness analysis of deep vein thrombosis prophylaxis in internal medicine patients. Thromb Res 1999;94(1):65-8.

41. Dentali F, Douketis JD, Gianni M, Lim W, Crowther MA. Meta-analysis: anticoagulant prophylaxis to prevent symptomatic venous thromboembolism in hospitalized medical patients. Ann Intern Med 2007;146(4):278-88.

45. Fontaine A, Mahe I, Bergmann JF, et al. Effectiveness of written guidelines on the appropriateness of thromboprophylaxis prescriptions for medical patients: a prospective randomized study. J Intern Med 2006;260(4):369-76.

48. Durieux P, Ravaud P, Dosquet P, Durocher A. [Effectiveness of clinical guideline implementation strategies: systematic review of systematic reviews]. Gastroenterol Clin Biol 2000;24(11):1018-25.

49. Thomson O'Brien MA, Oxman AD, Haynes RB, Davis DA, Freementle N, Harvey EL. Local opinion leaders: effects on professional practice and health care outcomes (Cochrane reviews). In: The Cochrane Library. Oxford: Update Software; 2001.

50. Durieux P, Nizard R, Ravaud P, Mounier N, Lepage E. A clinical decision support system for prevention of venous thromboembolism: effect on physician behavior. Jama 2000;283(21):2816-21.

51. Tooher R, Middleton P, Pham C, et al. A systematic review of strategies to improve prophylaxis for venous thromboembolism in hospitals. Annals of surgery 2005;241(3):397-415.

52. Grimshaw J, Campbell M, Eccles M, Steen N. Experimental and quasi-experimental designs for evaluating guideline implementation strategies. Fam Pract 2000;17 Suppl 1:S11-6.

53. Kucher N, Koo S, Quiroz R, et al. Electronic alerts to prevent venous thromboembolism among hospitalized patients. N Engl J Med 2005;352(10):969-77.

72. Perrier A, Roy PM, Sanchez O, et al. Multidetector-row computed tomography in suspected pulmonary embolism. N Engl J Med 2005;352(17):1760-8.

84. Auleley GR, Ravaud P, Giraudeau B, et al. Implementation of the Ottawa ankle rules in France. A multicenter randomized controlled trial. Jama 1997;277(24):1935-9.

85. Schulman S, Kearon C. Definition of major bleeding in clinical investigations of antihemostatic medicinal products in non-surgical patients. J Thromb Haemost 2005;3(4):692-4.

87. Donner A, Klar N. Statistical considerations in the design and analysis of community intervention trials. J Clin Epidemiol 1996;49(4):435-9.

88. Belch JJ, Lowe GD, Ward AG, Forbes CD, Prentice CR. Prevention of deep vein thrombosis in medical patients by low-dose heparin. Scott Med J 1981;26(2):115-7.

89. Dahan R, Houlbert D, Caulin C, et al. Prevention of deep vein thrombosis in elderly medical in-patients by a low molecular weight heparin: a randomized double-blind trial. Haemostasis 1986;16(2):159-64.

90. Gardlund B. Randomised, controlled trial of low-dose heparin for prevention of fatal pulmonary embolism in patients with infectious diseases. The Heparin Prophylaxis Study Group. Lancet 1996;347(9012):1357-61.

91. McGarry LJ, Thompson D. Retrospective database analysis of the prevention of venous thromboembolism with low-molecular-weight heparin in acutely III medical inpatients in community practice. Clinical therapeutics 2004;26(3):419-30.

92. Lederle FA, Sacks JM, Fiore L, et al. The prophylaxis of medical patients for thromboembolism pilot study. Am J Med 2006;119(1):54-9.

93. Leizorovicz A, Mismetti P. Preventing venous thromboembolism in medical patients. Circulation 2004;110(24 Suppl 1):IV13-9.

94. Klar N, Donner A. Current and future challenges in the design and analysis of cluster randomization trials. Stat Med 2001;20(24):3729-40.

95. Kerry SM, Bland JM. The intracluster correlation coefficient in cluster randomisation. Bmj 1998;316(7142):1455.

96. Kerry SM, Bland JM. Sample size in cluster randomisation. Bmj 1998;316(7130):549.

98. Stein PD, Fowler SE, Goodman LR, et al. Multidetector computed tomography for acute pulmonary embolism. N Engl J Med 2006;354(22):2317-27.

Appendix 1: Principal studies on preventive treatment in a medical setting.

| **Ref.** | **Medical disorder** | **Exclusion** | **Duration of follow-up** | **Intervention** | **N** | **DVT** | **PE +/- DVT** | **Fatal PE** | **Serious haemorrhage** |
| --- | --- | --- | --- | --- | --- | --- | --- | --- | --- |
| Belch et al, 1981  ^88^ | GHF – Respiratory infection | Age < 40 or > 80, major haemorrhagic risk, confined to bed > 2 d prior to admission, DVT or PE on admission | 14 d | UFH 5000 IUx3/d | 50 | ND | 0/50 (0%) | ND | ND |
|  |  |  |  | Placebo | 50 | ND | 2/50 (4%) | ND | ND |
| Dahan et al., 1986  ^89^ | GHF (NYHA II-IV), acute respiratory disorder | Age < 65 years, anticoagulant or antiaggregant treatment in progress, active haemorrhage or coagulation disorder, short hospitalised duration scheduled, allergy to iodine, thyroid pathology, autopsy not possible | 10 d | Enoxaparin 6000IUx1/d | 132 | ND | 1/132 (0.8%) | 1/132 (0.8%) | 1/132 (0.8%) |
|  |  |  |  | Placebo | 131 | ND | 3/131 (2.3%) | 3/131 (2.3%) | 3/131 (2.3%) |
| Gardlund et al. 1996  ^90^ | Acute infection | age < 55 years, anticoagulant treatment in progress, hospitalised again within 60 days after inclusion, active haemorrhage or coagulation disorder, renal or hepatic failure, HIV infection, end of life | 110 d | UFH 5000 IUx2/d | 5776 | ND | 3/5776 (0.1%) | 3/5776 (0.1%) | ND |
|  |  |  |  | Placebo | 5917 | ND | 12/5917 (0.2%) | 12/5917 (0.2%) | ND |
| Samama et al. 1999 ^28^ | GHF (NYHA II-IV), acute respiratory failure  or acute infection or acute rheumatic pathology | Age<40 years, pregnancy, lactation, woman of child-bearing age without contraception, stroke or major surgery within 3 months, contraindication to iodine-containing contrast media, thrombophilia, creatinine > 150µmol/L, intubation, HIV infection, uncontrolled HBP, active ulcer, endocarditis, haemorrhagic risk and coagulation disorder, history of HIT or hypersensitivity to heparin | 110 d | Enoxaparin 4000IUx1/d | 291 | 1/291  (0.3%) | 0/291(0%) | ND | 6/360 (1.7%) |
|  |  |  |  | Placebo | 288 | 2/288 (0.7%) | 3/288 (1%) | ND | 4/362 (1.1%) |
| Leizorovicz et al., 2004  ^30^ | Age ≥ 40 years, GHF (NYHA III-IV), acute or chronic respiratory failure, acute infection or acute rheumatic disease. | Age<40 or >80 years, weight < 45 or > 100 kg, history of VTE of at least 6 months, angiography contraindication, creatinine > 300 µmol/L, hepatic failure, uncontrolled HBP, haemorrhagic risk and coagulation disorder, history of HIT or hypersensitivity to heparin, anticoagulant or antiaggregant treatment in progress. | 90 d | Dalteparin 5000IUx1/d | 1740 | 5/1739  (0.3%) | 4/1740 (0.2%) | 0/1829 (0%) | 8/1856 (0.4%) |
|  |  |  |  | Placebo | 1759 | 11/1759(0.6%) | 5/1759 (0.3%) | 2/1807 (0.1%) | 0/1850 (0%) |
| McGarry et al., Clin Ther 2004  ^91^ | Hospitalisation in medicine for 6 days or more (retrospective analysis) for GHF, respiratory disorder, infectious pathology or cancer | Age < 40 years, active ulcer, uncontrolled hypertension, blood disease, HIV infection, tracheal or nasogastric intubation, pregnancy. | duration of hospitalisation | enoxaparin | 162 | 1.90% | 0/162 (0%) | ND | ND |
|  |  |  |  | no treatment | 3557 | 6.20% | 35/3557 (1%) | ND | ND |
| Mahé et al., 2005  ^101^ | GHF (NYHA III-IV), acute respiratory disorder, acute infection, cancer | Age<40 years, creatinine > 300 µmol/L, hepatic failure, uncontrolled HBP, active gastroduodenal ulcer, stroke or major surgery within 30 days, haemorrhagic risk and coagulation disorder, history of HIT or hypersensitivity to heparin, anticoagulant or antiaggregant treatment within 7 days, pregnancy. | 21 d | Nadroparin 7500IUx1/d | 1230 | ND | 10/1230 (1%) | 10/1230 (1%) | 1/1230 (0.1%) |
|  |  |  |  | Placebo | 1244 | ND | 17/1244 (1.4%) | 17/1244 (1.4%) | 3/1244 (0.3%) |
| Lederle et al., 2006  ^92^ | Scheduled hospitalisation in medicine for more than 3 days | Age<60 years, creatinine > 300 µmol/L, hepatic failure, uncontrolled HBP (> 220/110 mmHg), active gastroduodenal ulcer, MI or stroke or major or ophthalmological surgery within 30 days, endocarditis, haemorrhagic risk, anticoagulant treatment in progress or contraindication to anticoagulant treatment. | 90 d | Enoxaparin 4000IUx1/d | 140 | 4/140 (2.9%) | 1/140 (0.8%) | ND | 2/140 (1.4%) |
|  |  |  |  | Placebo | 140 | 6/140 (4.3%) | 3/140 (2.1%) | ND | 5/140 (3.6%) |
| Cohen et al., 2006  ^38^ | GHF (NYHA II-IV), acute respiratory disorder, infectious or inflammatory pathology. | Age<60 years, creatinine > 180 µmol/L, hepatic failure, brain metastases, stroke or recent ophthalmologic or brain or medullar surgery, endocarditis, haemorrhagic risk, anti-thrombotic treatment within 48 hours or indication of anti-thrombotic treatment, foreseeable intubation, life expectancy < 1 month. | 32 d | Fondaparinux 2.5mgx1/d | 429 | ND | 0/429 (0%) | 0/321 (0%) | ND |
|  |  |  |  | placebo | 420 | ND | 5/420 (1.2%) | 5/323 (1.2%) | ND |

Legend and abbreviations: ref.: reference; DVT: deep venous thrombosis; PE: pulmonary embolism; NA: not available; GHF: generalised heart failure; MI: myocardial infarction
