# Supplementary material for: Multifaceted Intervention to Prevent Venous Thromboembolism in Patients Hospitalized for Acute Medical Illness: A Multicenter Cluster-Randomized Trial
Source: PLoS One. 2016 May 26;11(5):e0154832. doi: 10.1371/journal.pone.0154832 (PMC4881951; doi:10.1371/journal.pone.0154832)
Supplement: S1 Statistical Analysis Plan — (PDF) [file pone.0154832.s005.pdf]

Prevention of Venous Thromboembolism Disease in Emergency Departments (PREvention de la maladie thromboembolique VEiNeuse dès les Urgences)

(PREVENU)

Statistical analysis plan

Last modified: July, 8th 2013

Author: Antoine Rachas

Co-authors: Gilles Chatellier, Jean-Marie Chrétien, Pierre Durieux, Pierre Hausfater, Guy Meyer, Dominique Mottier, Jeannot Schmidt, Pierre-Marie Roy

Table of contents

1. Introduction ... 3
2. Population ... 3
3. Missing data ... 3
4. Description of centers and patients ... 3
 4.1. Description of centers ... 3
 4.2. Description of patients ... 4
5. Primary end point analysis: thrombo-embolic and hemorrhagic events at 3 months. ... 5
 5.1. Description ... 5
 5.2. Effect of the intervention on the primary end point ... 5
  5.2.1. Regression models ... 5
  5.2.2. Decision rule ... 6
 5.3. Supplemental analyses ... 6
  5.3.1. Subgroups analyses ... 6
  5.3.2. Sensitivity analyses ... 6
6. Secondary clinical end points analysis ... 6
 6.1. Components of the primary end point ... 6
  6.1.1. Description ... 6
  6.1.2. Effect of the intervention on each component of the primary end point ... 7
 6.2. Mortality ... 7
  6.2.1. Description ... 7
  6.2.2. Effect of the intervention on all-cause mortality ... 7
 6.3. In-hospital outcomes ... 7
 6.4. Subgroups analyses ... 7
7. Prevention practices appropriateness ... 8
 7.1. Description ... 8
 7.2. Effect of the intervention on practices appropriateness ... 8
  7.2.1. Regression models ... 8
  7.2.2. Decision rule ... 10
 7.3. Supplemental analyses ... 10
  7.3.1. Subgroups analyses ... 10
  7.3.2. Sensitivity analyses ... 10
  7.3.1. Other analyses ... 10

1. Introduction

The statistical analysis plan was defined after database closure and before all statistical analysis. The scientific committee of the study and the statistician validated and signed it.

The statistician will be blinded for randomization group (coded 8 and 9) and centers' names. All the variables used for the analysis (ie, not in the case-report form), as well as the population, were defined by the PREVENU scientific committee.

2. Population

- As planned in the protocol, we will exclude:
  - patients hospitalized less than 48 hours (ie, who discharged the day or the day after their arrival).
  - patients with anticoagulant treatment at curative dose initiated during hospitalization AND continued 5 days or more, for another indication than a thrombo-embolic event. For patients with a long hospitalization, only the first 14 days were taken into account. Patients whose treatment was started during hospitalization but whose hospitalization was short and who discharged with the anticoagulant treatment, as well as the patients under treatment who died before day 14, were considered as treated from discharge time or death date to day 14.
- As also planned in the protocol,
  - for the prevention practices appropriateness analysis, subjects included during both pre- and post-intervention phases will be analyzed
  - for the primary and the secondary clinical endpoints analysis, only the subjects included during the post-intervention phase will be analyzed (no data about thrombo-embolic and hemorrhages during the pre-intervention phase)

3. Missing data

We will compare, by randomization group, the proportion and characteristics of patients whose data regarding outcomes was missing.

Patients whose data regarding the main outcome were missing will be excluded from the primary clinical endpoints analysis.

4. Description of centers and patients

4.1. Description of centers

- University hospital vs. general hospital
- Number of admissions in the emergency department per year
- Number of patients included in the study by week
- Level of prevention practice appropriateness in the pre-intervention phase
- Proportion of lost-to-follow-up

4.2. Description of patients

Following variables will be described and compared by randomization group and phase (pre or post-intervention)

- Demographic characteristics:
  - age
  - sex
- Health history & co-morbidities:
  - Previous thromboembolism
  - Chronic respiratory disease
  - Congestive heart failure
  - Active malignant condition
  - Chronic inflammatory disease
  - Surgery within 1 month
  - Fracture and/or orthopedic immobilization of a lower limb within 1 month
  - Hospitalization within 1 month
  - Current pregnancy or postpartum within 1 month (among women)
- Current treatment at admission:
  - Antiplatelet therapy
  - Hormonal treatment (with estrogenic compound)
- Renal function at admission
  - Creatininemia level
  - Creatininemia clearance (Simplified MDRD*)
    - ≥ 60 ml/min
    - 30 - 59 ml/min
    - 15 - 29 ml/min
    - < 15 ml/min

  * As the ethnic origin was not collected, all patients were considered as non-African
- Main hospitalization diagnosis (Respiratory failure; Acute coronary syndrome; Ischemic stroke and/or lower limb deficit; Heart failure; Infection sepsis; Acute rheumatologic condition; Inflammatory disease; Hemorrhage; Malignant condition; Neuropsychiatric disorder; Disorder induced by alcohol, toxic or drug; Digestive disorder; Cardio-vascular disorder; Respiratory disorder; Genital-urinary disorder; ORL and ophthalmologic disorder; Locomotor disorder; Hematologic disorder; Metabolic disorder; General status altered; Miscellaneous). Categories with very low number of patients will be grouped with 'Miscellaneous')
- Invasive procedures during hospitalization:
  - Surgery with general anesthesia
  - Indwelling central venous catheter or cardiac stimulator implantation
- Duration of hospitalization:
  - Median and quartiles — days
  - Mean and standard deviation – days.
  - >14 days

5. Primary end point analysis: thrombo-embolic and hemorrhagic events at 3 months.

5.1. Description

Incidence rate and its 95% confidence intervals, globally and by randomization group.

5.2. Effect of the intervention on the primary end point

5.2.1. Regression models

The OR for the primary end point will be estimated by using a mixed-effect logistic regression (SAS® 9.3 command: proc glimmix). We will measure the cluster effect by the intra-class correlation coefficient. The model will be the following (I denotes individuals, J denotes centers):

Model A1: `empty' model

$$Logit(P_{ij}) = \gamma_{00} + u_{0j}$$

where P is the probability of clinical event
I denotes individuals and J denotes centers
u_{0j} is a random effect, with $u_{0j} \sim N(0, \tau_{00})$

Model A2: only including the intervention

$$Logit(P_{ij}) = \gamma_{00}^{¥} + \gamma_{01}^{¥} \times intervention_j + u_{0j}$$

where P is the probability of clinical event
I denotes individuals and J denotes centers
u_{0j} is a random effect, with $u_{0j} \sim N(0, \tau_{00}^{¥})$

Model A3: also including confounders

$$Logit(P_{ij}) = \gamma_{00}^{¥*} + \gamma_{01}^{¥*} \times intervention_j + \sum_n (\gamma_{0n}^{¥*} \times W_{nj}) + \sum_k (\beta_k^{¥*} \times X_{kij}) + u_{0j}$$

where P is the probability of clinical event
I denotes individuals and J denotes centers
W_n are center-level confounders (n>1, as n=1 corresponds to $intervention_j$)
X_k are patient-level confounders
u_{0j} is a random effect, with $u_{0j} \sim N(0, \tau_{00}^{¥*})$

As the randomization was performed at centers' level, patient-level variables mentioned in '4.2 Description of patients' will be considered as potential confusion factors. Those with a p-value <0.20 when added in Model A2 will be included in Model A3. A stepwise selection will be performed until all covariables' p-values are <0.10.

Furthermore, as the randomization concerned only 27 units (the centers), we cannot exclude that confusion occurred by chance. Therefore, following the same method as for patient-level variables, we plan to adjust on potential center-level confounders:

- Number of admissions in the emergency department per year
- Number of inclusions by week

- University hospital (yes/no)

The hypothesis of linearity of the relation between each continuous variable (age, creatininemia clearance, duration of hospitalization, number of admissions in the emergency department per year, number of inclusions by week) and Logit(P_{ij}) will be verified. If this hypothesis cannot be assumed, the variables will be divided in quartiles, excepted creatininemia clearance, which will be divided as follows: ≥ 60 ml/min; 30 - 59 ml/min; 15 - 29 ml/min; < 15 ml/min.

Models A2 and A3 will allow estimating ORs. The expected percentage of patients matching the primary end point at 3 months is low (5%). We can then reasonably assume that the OR is close to the RR. Both crude and adjusted OR and their 95% confidence intervals will be estimated.

5.2.2. Decision rule

The intervention will be considered as effective on the primary end point if the Wald test's p-value of $\gamma_{01}^{\Psi*}$ (Model A3) is <0.05 (ie, the adjusted OR of the intervention is significantly different from 1).

5.3. Supplemental analyses

5.3.1. Subgroups analyses

The incidence rate and OR will be estimated with the same methods in the following subgroups:

- by age (≤ or > 75 y.o.)
- according to whether or not a treatment was recommended
- by level of prevention practice appropriateness in the pre-intervention phase (divided in quartiles). This is a center-level variable.

5.3.2. Sensitivity analyses

We will perform the same analysis

- using two other definitions of the primary endpoint:
  - Not considering unexplained sudden deaths as thrombo-embolic events
  - Considering unexplained deaths for which we did not know whether or not they were sudden as thrombo-embolic events
- after exclusion of the centers with more than 10% of patients lost-to-follow-up
- after exclusion of the center with a computerized reminder of appropriate practices (to be done at the end of the analysis because it needs to reveal centers' names)

6. Secondary clinical end points analysis

6.1. Components of the primary end point

6.1.1. Description

Incidence rates of the components of the primary end point and their 95% confidence intervals, globally and by randomization group, namely:

For thrombo-embolic events:

- All thromboembolic events
- Pulmonary embolism (including fatal pulmonary embolism)
- Proximal deep venous thrombosis

- Distal deep venous thrombosis
- Unexplained sudden death

For hemorrhagic events:

- All major bleedings, including fatal bleeding
- Fatal hemorrhage

6.1.2. Effect of the intervention on each component of the primary end point

Following a similar method as for the primary end point analysis, we will study following outcomes:

- All thromboembolic events
- All major bleedings, including fatal bleeding

The confusion factors will be the same than for the primary end point.

6.2. Mortality

6.2.1. Description

Incidence rates of the following events and their 95% confidence intervals, globally and by randomization group, will be estimated:

- All-cause mortality
- Fatal pulmonary embolism
- Unexplained sudden death
- Fatal hemorrhage
- Death unrelated to PE or hemorrhage
- Death with insufficient information

6.2.2. Effect of the intervention on all-cause mortality

Following a similar method as for the primary end point analysis, we will study the effect of the intervention on all-cause mortality. The potential confusion factors will be selected with the same method.

6.3. In-hospital outcomes

To allow comparison with other studies, which were limited to in-hospital outcomes, we will perform a similar analysis on following outcomes:

- In-hospital thrombo-embolic and hemorrhagic events
- In-hospital thrombo-embolic events
- In-hospital hemorrhagic events
- In-hospital mortality (all causes)

The confusion factors will be the same than for the primary end point.

6.4. Subgroups analyses

The descriptive analysis of the components of the primary end point and of mortality will be performed in the following subgroups:

- by age (≤ or > 75 y.o.)

- according to whether or not a treatment was recommended
- by level of prevention practice appropriateness in the pre-intervention phase (divided in quartiles). This is a center-level variable.

The effect of the intervention will be analyzed in these subgroups for:

- All thromboembolic events
- All major bleedings, including fatal bleeding
- All-cause mortality

7. Prevention practices appropriateness

7.1. Description

The level of appropriateness will be described by randomization group and phase (pre or post-intervention):

- Appropriate prevention practices
  - All
  - Treatment recommended and received before day 5, right dosage and duration (5 days or more)
  - Treatment not recommended and not received
  - Indication against treatment and treatment not received
- Inappropriate prevention practices
  - All
  - Treatment recommended but not received
  - Treatment recommended, received, but wrong dosage, starting time or duration
  - Treatment not recommended but received before day 5, right dosage and duration (5 days or more)
  - Treatment not recommended but received at wrong dosage or duration
  - Indication against treatment and treatment received

7.2. Effect of the intervention on practices appropriateness

7.2.1. Regression models

The improvement of prevention practice appropriateness (appropriate vs. inappropriate) between pre- and post-intervention phases will be compared between intervention and control groups by using a mixed-effect logistic regression (SAS® 9.3 command: proc glimmix). We will measure the cluster effect by the intra-class correlation coefficient. The model will be built as follows.

Model B1: `empty' model

$$Logit(P'_{ij}) = \gamma_{00}^{\P} + \gamma_{10} \times phase_{ij} + u_{0j} + u_{1j}$$

where P' is the probability of appropriate practice

I denotes individuals and J denotes centers

u_{0j} *and* u_{1j} are random effects, with $u_{0j} \sim N(0, \tau_{00}^{\P})$ and $u_{1j} \sim N(0, \tau_{11}^{\P})$

Model B2: including the intervention

$$Logit(P'_{ij}) = \gamma_{00}^{*} + \gamma_{01}^{*} \times intervention_j + \gamma_{10}^{*} \times phase_{ij} + \gamma_{11}^{*} \times intervention_j \times phase_{ij} + u_{0j} + u_{1j}$$

where P' is the probability of appropriate practice

I denotes individuals and J denotes centers

u_{0j} *and* u_{1j} are random effects, with $u_{0j} \sim N(0, \tau_{00}^{*})$ and $u_{1j} \sim N(0, \tau_{11}^{*})$

Here we consider γ_{11}^{*} as a randomly varying slope. If τ_{11}^{*} does not significantly differ from 0, γ_{11}^{*} will be considered as a non-randomly varying slope (simple interaction).

Model B3: also including confounders

$$Logit(P'_{ij}) = \gamma_{00}^{**} + \gamma_{01}^{**} \times intervention_j + \sum_n (\gamma_{0n}^{**} \times W_{nj}) + \gamma_{10}^{**} \times phase_{ij} + \gamma_{11}^{**} \times intervention_j \times phase_{ij} + \sum_k (\beta_k^{**} \times X_{kij}) + u_{0j} + u_{1j}$$

where P' is the probability of appropriate practice

I denotes individuals and J denotes centers

W_n are center-level confounders (n>1, as n=1 corresponds to $intervention_j$)

X_k are patient-level confounders

u_{0j} *and* u_{1j} are random effects, with $u_{0j} \sim N(0, \tau_{00}^{**})$ and $u_{1j} \sim N(0, \tau_{11}^{**})$

As the randomization was performed at centers' level, patient-level variables mentioned in '4.2 Description of patients' will be considered as potential confusion factors. Those with a p-value <0.20 when added in Model B2 will be included in Model B3. A stepwise selection will be performed until all covariables' p-values are <0.10.

Furthermore, as the randomization concerned only 27 units (the centers), we cannot exclude that confusion occurred by chance. Therefore, following the same method as for patient-level variables, we plan to adjust on potential center-level confounders:

- Number of admissions in the emergency department per year
- Number of inclusions by week
- University hospital (yes/no)

The hypothesis of linearity of the relation between each continuous variable (age, creatininemia clearance, duration of hospitalization, number of admissions in the emergency department per year, number of inclusions by week) and Logit(P'_{ij}) will be verified. If this hypothesis cannot be assumed, the variables will be divided in quartiles, excepted creatininemia clearance, which will be divided as follows: ≥ 60 ml/min; 30 - 59 ml/min; 15 - 29 ml/min; < 15 ml/min.

7.2.2. Decision rule

The intervention will be considered as having modified prevention practice appropriateness if the Wald test's p-value of γ_{11}^{**} (Model B3) is <0.05.

7.3. Supplemental analyses

7.3.1. Subgroups analyses

The incidence rate and OR will be estimated with the same methods according to whether or not a treatment was recommended.

7.3.2. Sensitivity analyses

We will perform the same analysis with a more restrictive definition of appropriateness: appropriate preventive treatment initiated the day or the day after the arrival.

7.3.1. Other analyses

We will compare the median time to initiate treatment (limited to 14 days or duration of hospitalization), in patients with an appropriate treatment with a Wilcoxon signed-rank test.
