# Supplementary material for: Multifaceted Intervention to Prevent Venous Thromboembolism in Patients Hospitalized for Acute Medical Illness: A Multicenter Cluster-Randomized Trial
Source: PLoS One. 2016 May 26;11(5):e0154832. doi: 10.1371/journal.pone.0154832 (PMC4881951; doi:10.1371/journal.pone.0154832)
Supplement: S1 Table — (DOC) [file pone.0154832.s006.doc]

| S1 Table. Baseline characteristics of patients included during the intervention period by lost to follow-up status. | | | | | | | | | | | | |
| --- | --- | --- | --- | --- | --- | --- | --- | --- | --- | --- | --- | --- |
|  |  | Intervention group | | | | |  | Control group | | | | |
|  |  | Lost to follow-up | | | |  |  | Lost to follow-up | | | |  |
|  |  | Yes | | No | | p |  | Yes | | No | | p |
|  |  | N = 291 | | N = 8068 | |  |  | N = 300 | | N = 6692 | |  |
| **Demographic characteristics** | |  |  |  |  |  |  |  |  |  |  |  |
|  | Male— n (%) | 151 | (51.9) | 3985 | (49.4) | 0.40 |  | 149 | (49.7) | 3387 | (50.6) | 0.75 |
|  | Median age— y (IQR) | 72 | (56 - 85) | 74 | (59 - 83) | 0.30 |  | 73.5 | (57 - 84) | 73 | (58 - 82) | 0.54 |
| **Health history & co-morbidities** | |  |  |  |  |  |  |  |  |  |  |  |
|  | Previous thromboembolism— n (%) | 14 | (4.8) | 473 | (5.9) | 0.45 |  | 15 | (5.0) | 433 | (6.5) | 0.31 |
|  | Chronic respiratory disease — n (%) | 32 | (11.0) | 1303 | (16.2) | 0.018 |  | 50 | (16.7) | 1176 | (17.6) | 0.70 |
|  | Congestive heart failure — n (%) | 29 | (10.0) | 820 | (10.2) | 0.92 |  | 44 | (14.7) | 994 | (14.9) | 0.92 |
|  | Chronic inflammatory disease — n (%) | 5 | (1.7) | 288 | (3.6) | 0.093 |  | 4 | (1.3) | 259 | (3.9) | 0.024 |
|  | Active malignant condition — n (%) | 45 | (15.5) | 1174 | (14.6) | 0.67 |  | 58 | (19.3) | 963 | (14.4) | 0.018 |
|  | Surgery within 1 month — n (%) | 8 | (2.8) | 168 | (2.1) | 0.43 |  | 4 | (1.3) | 109 | (1.6) | 0.99 |
|  | Hospitalization within 1 month — no. (%) | 27 | (9.3) | 726 | (9.0) | 0.86 |  | 31 | (10.3) | 645 | (9.7) | 0.69 |
| **Current treatment at admission** | |  |  |  |  |  |  |  |  |  |  |  |
|  | Antiplatelet therapy — n (%) | 81 | (27.9) | 2804 | (34.8) | 0.016 |  | 80 | (26.8) | 2194 | (32.8) | 0.031 |
|  | Hormonal treatment (with estrogenic compound) — n (%) | 1 | (0.3) | 50 | (0.6) | 0.99 |  | 2 | (0.7) | 37 | (0.6) | 0.68 |
| **Renal function at admission** | |  |  |  |  |  |  |  |  |  |  |  |
|  | Median creatinin — μmol/l (IQR) | 78 | (64 - 104) | 78 | (63 - 102) | 0.66 |  | 84 | (68 - 107) | 86 | (69 - 114) | 0.42 |
|  | Median creatinin clearance* — ml/min (IQR) | 79 | (56.3 - 98.4) | 78.7 | (57.1 - 99.7) | 0.87 |  | 70.6 | (52.3 - 92.2) | 71.7 | (50.2 - 91.9) | 0.67 |
|  | Creatinin clearance* |  |  |  |  |  |  |  |  |  |  |  |
|  | ≥ 60 ml/min — n (%) | 173 | (71.2) | 5400 | (72.0) |  |  | 183 | (67.5) | 3900 | (64.4) |  |
|  | 30-59 ml/min – n (%) | 47 | (19.3) | 1645 | (21.9) |  |  | 64 | (23.6) | 1613 | (26.6) |  |
|  | 15-30 ml/min – n (%) | 13 | (5.3) | 335 | (4.5) |  |  | 15 | (5.5) | 355 | (5.9) |  |
|  | <15 ml/min – n (%) | 10 | (4.1) | 120 | (1.6) | 0.034 |  | 9 | (3.3) | 186 | (3.1) | 0.71 |
| **Main reason for admission** | |  |  |  |  |  |  |  |  |  |  |  |
|  | with high risk of TE event — n (%) | 90 | (31.3) | 2465 | (30.7) |  |  | 95 | (32.0) | 2197 | (33.0) |  |
|  | with moderate risk of TE event — n (%) | 87 | (30.2) | 2295 | (28.6) |  |  | 76 | (25.6) | 1840 | (27.6) |  |
|  | with low risk of TE event — n (%) | 100 | (34.7) | 2937 | (36.6) |  |  | 114 | (38.4) | 2323 | (34.9) |  |
|  | with contraindication to anticoagulant — n (%) | 11 | (3.8) | 325 | (4.1) | 0.90 |  | 12 | (4.0) | 298 | (4.5) | 0.65 |
| **Procedures during hospitalization** | |  |  |  |  |  |  |  |  |  |  |  |
|  | Surgery (general or regional anesthesia)— n (%) | 6 | (2.1) | 277 | (3.4) | 0.21 |  | 11 | (3.7) | 309 | (4.6) | 0.47 |
|  | Indwelling central venous catheter or cardiac stimulator implantation — n (%) | 1 | (0.3) | 72 | (0.9) | 0.52 |  | 2 | (0.7) | 88 | (1.3) | 0.59 |
| **Duration of hospitalization** | |  |  |  |  |  |  |  |  |  |  |  |
|  | Median — days (IQR) | 7 | (4 - 14) | 7 | (4 - 13) | 0.81 |  | 7 | (4 - 13) | 8 | (4 - 14) | 0.028 |
|  | >14 days — n (%) | 71 | (24.4) | 1573 | (19.5) | 0.039 |  | 64 | (21.3) | 1561 | (23.3) | 0.42 |

## p-values are based on χ² (or Fisher exact) tests for qualitative data and Mann-Withney tests for quantitative data. These tests do not account for cluster effect. *Creatinin clearance calculated by simplified MDRD formula, considering all patients were white
