# Supplementary material for: Multifaceted Intervention to Prevent Venous Thromboembolism in Patients Hospitalized for Acute Medical Illness: A Multicenter Cluster-Randomized Trial
Source: PLoS One. 2016 May 26;11(5):e0154832. doi: 10.1371/journal.pone.0154832 (PMC4881951; doi:10.1371/journal.pone.0154832)
Supplement: S2 Table — (DOC) [file pone.0154832.s007.doc]

| S2 Table. Subgroups analysis of clinical events | | | | | | | | | | | | | | |
| --- | --- | --- | --- | --- | --- | --- | --- | --- | --- | --- | --- | --- | --- | --- |
|  |  | Intervention group | | Control group | | No. of subjects included in models | OR (95% CI) adjusted for cluster effect only* | | p |  | OR (95% CI) adjusted for cluster and fixed effects** | | p |  |
|  |  | N = 8359 | | N = 6992 | |  |  |
| ≤ 75 years-old | | 4501 |  | 3912 |  |  |  |  |  |  |  |  |  |  |
|  | Thromboembolic event or major bleeding — no. (%) | 105 | (2.4) | 87 | (2.3) | 7287 | 1.13 | (0.77 - 1.66) | 0.52 |  | 1.17 | (0.79 - 1.71) | 0.43 |  |
|  | Thromboembolic event — no. (%) | 60 | (1.4) | 48 | (1.3) | 7287 | 1.16 | (0.65 - 2.07) | 0.61 |  | 1.18 | (0.65 - 2.13) | 0.59 |  |
|  | Major bleeding (including fatal) — no. (%) | 45 | (1.0) | 39 | (1.0) | 7287 | 1.04 | (0.50 - 2.17) | 0.92 |  | 1.12 | (0.53 - 2.34) | 0.77 |  |
|  | Death — no. (%) | 344 | (7.7) | 282 | (7.3) | 7432 | 1.05 | (0.80 - 1.38) | 0.70 |  | 1.15 | (0.90 - 1.47) | 0.25 |  |
| >75 years-old | | 3858 |  | 3080 |  |  |  |  |  |  |  |  |  |  |
|  | Thromboembolic event or major bleeding — no. (%) | 145 | (3.9) | 127 | (4.3) | 6170 | 0.88 | (0.63 - 1.22) | 0.44 |  | 0.92 | (0.67 - 1.28) | 0.63 |  |
|  | Thromboembolic event — no. (%) | 90 | (2.4) | 80 | -2.7 | 6170 | 0.87 | (0.57 - 1.31) | 0.50 |  | 0.88 | (0.60 - 1.31) | 0.54 |  |
|  | Major bleeding (including fatal) — no. (%) | 55 | (1.5) | 47 | (1.6) | 6170 | 0.9 | (0.57 - 1.43) | 0.67 |  | 1.00 | (0.61 - 1.64) | 0.99 |  |
|  | Death — no. (%) | 596 | (15.5) | 482 | (15.9) | 6322 | 0.92 | (0.74 - 1.15) | 0.47 |  | 0.95 | (0.75 - 1.20) | 0.66 |  |
| Treatment not recommended | | 4137 |  | 3411 |  |  |  |  |  |  |  |  |  |  |
|  | Thromboembolic event or major bleeding — no. (%) | 107 | (2.7) | 92 | (2.8) | 6514 | 0.97 | (0.68 - 1.37) | 0.86 |  | 0.99 | (0.69 - 1.41) | 0.95 |  |
|  | Thromboembolic event — no. (%) | 64 | (1.6) | 51 | (1.6) | 6514 | 1.02 | (0.65 - 1.60) | 0.94 |  | 0.99 | (0.63 - 1.56) | 0.97 |  |
|  | Major bleeding (including fatal) — no. (%) | 43 | (1.1) | 41 | (1.3) | 6514 | 0.84 | (0.43 - 1.65) | 0.62 |  | 0.90 | (0.45 - 1.81) | 0.78 |  |
|  | Death — no. (%) | 368 | (9.0) | 301 | (9.0) | 6652 | 1.00 | (0.74 - 1.35) | 0.99 |  | 1.04 | (0.76 - 1.41) | 0.82 |  |
| Treatment recommended | | 4222 |  | 3581 |  |  |  |  |  |  |  |  |  |  |
|  | Thromboembolic event or major bleeding — no. (%) | 143 | (3.5) | 122 | (3.6) | 6943 | 1.05 | (0.72 - 1.54) | 0.81 |  | 1.08 | (0.75 - 1.54) | 0.69 |  |
|  | Thromboembolic event — no. (%) | 86 | (2.1) | 77 | -2.2 | 6943 | 1.03 | (0.63 - 1.67) | 0.91 |  | 1.03 | (0.66 - 1.61) | 0.90 |  |
|  | Major bleeding (including fatal) — no. (%) | 57 | (1.4) | 45 | (1.3) | 6943 | 1.07 | (0.64 - 1.78) | 0.79 |  | 1.15 | (0.67 - 1.98) | 0.61 |  |
|  | Death — no. (%) | 572 | (13.6) | 463 | (13.1) | 7102 | 0.94 | (0.72 - 1.22) | 0.63 |  | 0.97 | (0.74 - 1.25) | 0.79 |  |
| Centers of the 1st quartile of pre-intervention adequacy | | 1039 |  | 1860 |  |  |  |  |  |  |  |  |  |  |
|  | Thromboembolic event or major bleeding — no. (%) | 38 | (3.9) | 53 | (2.9) | 2675 | 1.35 | (0.87 - 2.07) | 0.18 |  | 1.49 | (0.95 - 2.33) | 0.083 |  |
|  | Thromboembolic event — no. (%) | 29 | (3.0) | 33 | (1.8) | 2675 | 1.60 | (0.97 - 2.65) | 0.07 |  | 1.79 | (1.07 - 3.02) | 0.028 |  |
|  | Major bleeding (including fatal) — no. (%) | 9 | (0.9) | 20 | (1.1) | 2675 | 0.9 | (0.31 - 2.29) | 0.74 |  | 0.89 | (0.37 - 2.13) | 0.80 |  |
|  | Death — no. (%) | 148 | (14.5) | 213 | (11.6) | 2751 | 1.27 | (1.01 - 1.59) | 0.044 |  | 1.54 | (1.19 - 2.00) | 0.001 |  |
| Centers of the 2nd quartile of pre-intervention adequacy | | 2676 |  | 1483 |  |  |  |  |  |  |  |  |  |  |
|  | Thromboembolic event or major bleeding — no. (%) | 74 | (2.9) | 48 | (3.4) | 3504 | 0.78 | (0.32 - 1.86) | 0.57 |  | 0.95 | (0.63 - 1.44) | 0.83 |  |
|  | Thromboembolic event — no. (%) | 35 | (1.4) | 33 | (2.3) | 3504 | 0.5 | (0.28 - 0.97) | 0.04 |  | 0.59 | (0.35 – 1.00) | 0.049 |  |
|  | Major bleeding (including fatal) — no. (%) | 39 | (1.5) | 15 | (1.1) | 3504 | 1.1 | (0.29 - 4.01) | 0.92 |  | 1.56 | (0.51 - 4.78) | 0.43 |  |
|  | Death — no. (%) | 271 | (10.2) | 142 | (9.7) | 3592 | 0.77 | (0.28 - 2.14) | 0.61 |  | 0.75 | (0.28 - 2.01) | 0.57 |  |
| Centers of the 3rd quartile of pre-intervention adequacy | | 1619 |  | 2498 |  |  |  |  |  |  |  |  |  |  |
|  | Thromboembolic event or major bleeding — no. (%) | 54 | (3.5) | 82 | (3.5) | 3374 | 1.11 | (0.66 - 1.87) | 0.69 |  | 1.12 | (0.72 - 1.76) | 0.62 |  |
|  | Thromboembolic event — no. (%) | 38 | (2.4) | 40 | (1.8) | 3374 | 1.65 | (0.79 - 3.41) | 0.18 |  | 1.35 | (0.77 - 2.37) | 0.30 |  |
|  | Major bleeding (including fatal) — no. (%) | 16 | (1.0) | 42 | (1.8) | 3374 | 0.66 | (0.35 - 1.22) | 0.18 |  | 0.80 | (0.38 - 1.66) | 0.54 |  |
|  | Death — no. (%) | 200 | (12.4) | 291 | (11.9) | 3431 | 1.04 | (0.64 - 1.68) | 0.88 |  | 1.06 | (0.67 - 1.67) | 0.80 |  |
| Centers of the 4th quartile of pre-intervention adequacy | | 3025 |  | 1151 |  |  |  |  |  |  |  |  |  |  |
|  | Thromboembolic event or major bleeding — no. (%) | 84 | (2.8) | 31 | (2.8) | 3904 | 1.06 | (0.64 - 1.75) | 0.83 |  | 1.19 | (0.72 - 1.96) | 0.50 |  |
|  | Thromboembolic event — no. (%) | 48 | (1.6) | 22 | (2.0) | 3904 | 0.81 | (0.39 - 1.68) | 0.58 |  | 0.88 | (0.40 - 1.94) | 0.74 |  |
|  | Major bleeding (including fatal) — no. (%) | 36 | (1.2) | 9 | (0.8) | 3904 | 1.88 | (0.69 - 5.16) | 0.22 |  | 2.26 | (0.82 - 6.23) | 0.11 |  |
|  | Death — no. (%) | 321 | (10.7) | 118 | (10.4) | 3980 | 0.96 | (0.76 - 1.20) | 0.69 |  | 0.98 | (0.76 - 1.25) | 0.86 |  |

*OR from mixed logistic regression including center as random intercept
** fixed effects were:
- for thromboembolic event and/or major bleeding: age, sex, history of active malignant condition, hospitalization within 1 month, renal function at admission, main acute medical condition, surgery (general or regional anesthesia), indwelling central venous catheter or cardiac stimulator implantation, length of hospitalization, university hospital
- for mortality: same factors, plus history of previous thromboembolism, history of congestive heart failure, antiplatelet therapy.
