# Supplementary material for: Multifaceted Intervention to Prevent Venous Thromboembolism in Patients Hospitalized for Acute Medical Illness: A Multicenter Cluster-Randomized Trial
Source: PLoS One. 2016 May 26;11(5):e0154832. doi: 10.1371/journal.pone.0154832 (PMC4881951; doi:10.1371/journal.pone.0154832)
Supplement: S3 Table — (DOC) [file pone.0154832.s008.doc]

| S3 Table. Sensitivity analyses of the main outcome | | | | | | | | | | | | | | |
| --- | --- | --- | --- | --- | --- | --- | --- | --- | --- | --- | --- | --- | --- | --- |
|  |  |  | Intervention group | | Control  group | | No. of subjects included in models | OR (95% CI) adjusted for cluster effect only* | | p | OR (95% CI) adjusted for cluster and fixed effects** | | p |  |
| Different definitions of the main outcome | | |  |  |  |  |  |  |  |  |  |  |  |  |
|  |  | Thromboembolic event or major bleeding not considering unexplained sudden deaths as TE events — no. (%) | 179/8068 | (2.2) | 156/6692 | (2.3) | 13457 | 0.96 | (0.67 - 1.39) | 0.85 | 1.01 | (0.71 - 1.43) | 0.96 |  |
|  |  | Thromboembolic event or major bleeding considering unexplained deaths for which we did not know whether or not they were sudden as TE events — no. (%) | 267/8085 | (3.3) | 233/6711 | (3.5) | 13489 | 1.00 | (0.76 - 1.30) | 0.97 | 1.01 | (0.78 - 1.30) | 0.93 |  |
| Exclusion of the centers with >10% lost to follow-up (n=3/27) | | |  | |  | |  |  |  |  |  |  |  |  |
|  | Thromboembolic event or major bleeding — no. (%) | | 241/7583 | (3.2) | 210/6586 | (3.2) | 13141 | 1.02 | (0.77 - 1.34) | 0.90 | 1.06 | (0.81 - 1.38) | 0.68 |  |

## *OR from mixed logistic regression including center as random intercept ** fixed effects were: - for thromboembolic event and/or major bleeding: age, sex, history of active malignant condition, hospitalization within 1 month, renal function at admission, main acute medical condition, surgery (general or regional anesthesia), indwelling central venous catheter or cardiac stimulator implantation, length of hospitalization, university hospital - for mortality: same factors, plus history of previous thromboembolism, history of congestive heart failure, antiplatelet therapy
