# Supplementary material for: Multifaceted Intervention to Prevent Venous Thromboembolism in Patients Hospitalized for Acute Medical Illness: A Multicenter Cluster-Randomized Trial
Source: PLoS One. 2016 May 26;11(5):e0154832. doi: 10.1371/journal.pone.0154832 (PMC4881951; doi:10.1371/journal.pone.0154832)
Supplement: S4 Table — (DOC) [file pone.0154832.s009.doc]

| S4 Table. In-hospital outcomes | | | | | | | | | | | | | |
| --- | --- | --- | --- | --- | --- | --- | --- | --- | --- | --- | --- | --- | --- |
|  |  | Intervention group | |  | Control group | | No. of subjects included in models | OR (95% CI), adjusted for cluster effect only* | | p | OR (95% CI), adjusted for cluster and fixed effects** | | p |
| Thromboembolic event or major bleeding — no. (%) | | 117/8347 | (1.4) |  | 107/6978 | (1.5) | 13942 | 1.01 | (0.67 - 1.52) | 0.97 | 1.07 | (0.72 - 1.58) | 0.74 |
| First event: | |  |  |  |  |  |  |  |  |  |  |  |  |
| Thromboembolic event — no. (%) | | 60/8347 | (0.7) |  | 62/6981 | (0.9) | 13945 | 0.89 | (0.51 - 1.55) | 0.68 | 0.94 | (0.55 - 1.62) | 0.83 |
| Major bleeding (including fatal) — no. (%) | | 57/8349 | (0.7) |  | 45/6981 | (0.6) | 13947 | 1.12 | (0.59 - 2.11) | 0.72 | 1.19 | (0.63 - 2.25) | 0.59 |
| Death — no. (%) | | 436/8359 | (5.2) |  | 408/6991 | (5.8) | 13906 | 0.90 | (0.68 - 1.19) | 0.46 | 0.97 | (0.73 - 1.29) | 0.85 |

*OR from mixed logistic regression including a random intercept at centers’ level
** Fixed effects were:
- for thromboembolic event and/or major bleeding: age, sex, history of active malignant condition, hospitalization within 1 month, renal function at admission, main acute medical condition, surgery (general or regional anesthesia), indwelling central venous catheter or cardiac stimulator implantation, length of hospitalization, university hospital
- for mortality: same factors, plus history of previous thromboembolism, history of congestive heart failure, antiplatelet therapy
