# Supplementary material for: Multifaceted Intervention to Prevent Venous Thromboembolism in Patients Hospitalized for Acute Medical Illness: A Multicenter Cluster-Randomized Trial
Source: PLoS One. 2016 May 26;11(5):e0154832. doi: 10.1371/journal.pone.0154832 (PMC4881951; doi:10.1371/journal.pone.0154832)
Supplement: S5 Table — (DOC) [file pone.0154832.s010.doc]

| S5 Table. Thromboprophylaxis practices adequacy according to whether a preventive anticoagulant treatment was recommended | | | | | | | | | | | | | | | | | | | | | | | | | | | | | | | | | | | | | | | | | | | | | | | | |  |
| --- | --- | --- | --- | --- | --- | --- | --- | --- | --- | --- | --- | --- | --- | --- | --- | --- | --- | --- | --- | --- | --- | --- | --- | --- | --- | --- | --- | --- | --- | --- | --- | --- | --- | --- | --- | --- | --- | --- | --- | --- | --- | --- | --- | --- | --- | --- | --- | --- | --- |
|  |  |  | Intervention group | | | | | | | | | | | | | | | | | |  | | Control group | | | | | | | | | | | | | | | | | |  | | Adjusted difference in change (95% CI), percentage points*† | | | p value | | | |
|  |  |  | Period | | | | | | | | | | | |  | | Adjusted absolute change, %*§ | | | |  | | Period | | | | | | | | | | | |  | | Adjusted absolute change, %*§ | | | |  | |
|  |  | Pre-intervention | | | | |  | | Intervention | | | |  | | |  | | |  | | | Pre-intervention | | | | |  | | Intervention | | | |  | | |  | | |  | | |  | | |  | |  | | |
| **All patients** |  |  | | |  | |  | |  | |  | |  | | |  | | |  | | |  | | |  | |  | |  | |  | |  | | |  | | |  | | |  | | |  | |  | | |
| Adequate practices — no. (%) | 373/712 | | | (52.4) | |  | | 4254/8359 | | (50.9) | |  | | -1.4 | | | |  | | 339/690 | | | | (49.1) | |  | | 3413/6992 | | (48.8) | |  | | -0.2 | | | |  | | -1.2 (-6.6 to 4.3) | | | | 0.68 | | | |  | |
| **Treatment recommended** | | | | | | | | | | | | |  | | |  | | |  | | |  | | |  | |  | |  | |  | |  | | |  | | |  | | |  | | |  | |  | | |
| Adequate practices — no. (%) | 73/319 | | | (22.9) | |  | | 1474/4222 | | (34.9) | |  | | 9.5 | | | |  | | 81/318 | | | | (25.5) | |  | | 1094/3581 | | (30.6) | |  | | 6.5 | | | |  | | 3.0 (-3.7 to 9.6) | | | | 0.38 | | | |  | |
| **Treatment not recommended** |  |  | | |  | |  | |  | |  | |  | | |  | | |  | | |  | | |  | |  | |  | |  | |  | | |  | | |  | | |  | | |  | |  | | |
| Adequate practices — no. (%) | 300/393 | | | (76.3) | |  | | 2780/4137 | | (67.2) | |  | | -7.2 | | | |  | | 258/372 | | | | (69.4) | |  | | 2319/3411 | | (68.0) | |  | | -1.4 | | | |  | | -5.8 (-12.3 to 0.7) | | | | 0.082 | | | |  | |

* Adjusted for cluster effect, age, history of chronic respiratory disease, history of active malignant condition, antiplatelet therapy, surgery (general or regional anesthesia), indwelling central venous catheter or cardiac stimulator implantation, length of hospitalization.
† Difference in absolute change of adequacy between the intervention and control groups.
§ Adjusted absolute change in the frequency of adequate prevention practice between the pre-intervention and intervention periods.
