# Supplementary material for: Multifaceted Intervention to Prevent Venous Thromboembolism in Patients Hospitalized for Acute Medical Illness: A Multicenter Cluster-Randomized Trial
Source: PLoS One. 2016 May 26;11(5):e0154832. doi: 10.1371/journal.pone.0154832 (PMC4881951; doi:10.1371/journal.pone.0154832)
Supplement: S6 Table — (DOC) [file pone.0154832.s011.doc]

| S6 Table. Thromboprophylaxis practices adequacy and delay of prescription | | | | | | | | | | | | | | | | | | | | | | |
| --- | --- | --- | --- | --- | --- | --- | --- | --- | --- | --- | --- | --- | --- | --- | --- | --- | --- | --- | --- | --- | --- | --- |
|  | |  |  | Intervention group | | | | | | |  | Control group | | | | | | |  | Adjusted difference in change  (95% CI), percentage points*† | p value |  |
|  | |  |  | Period | | | | |  | Adjusted absolute change, %*§ |  | Period | | | | |  | Adjusted absolute change, %*§ |  |  |  |  |
|  | |  |  | Pre-intervention | |  | Intervention | |  |  |  | Pre-intervention | |  | Intervention | |  |  |  |  |  |  |
|  | |  |  |  |  |  |  |  |  |  |  |  |  |  |  |  |  |  |  |  |  |  |
| Adequate practices — no. (%) | | |  | 373/712 | (52.4) |  | 4254/8359 | (50.9) |  | -1.4 |  | 339/690 | (49.1) |  | 3413/6992 | (48.8) |  | -0.2 |  | -1.2 (-6.6 to 4.3) | 0.68 |  |
| Adequate practices and prescription at J1 or J2 — no. (%) | | |  | 360/712 | (50.6) |  | 3999/8359 | (47.8) |  | -2.4 |  | 322/690 | (46.7) |  | 3191/6992 | (45.6) |  | -0.8 |  | -1.6 (-7.0 to 3.8) | 0.56 |  |
|  |  | |  |  |  |  |  |  |  |  |  |  |  |  |  |  |  |  |  |  |  |  |

* Adjusted for cluster effect, age, history of chronic respiratory disease, active malignant condition, antiplatelet therapy, surgery (general or regional anesthesia), indwelling central venous catheter or cardiac stimulator implantation, length of hospitalization.
† Difference in absolute change of adequacy between the intervention and control groups.
§ Adjusted absolute change in the frequency of adequate prevention practice between the pre-intervention and intervention periods.
