# Supplementary material for: Multifaceted Intervention to Prevent Venous Thromboembolism in Patients Hospitalized for Acute Medical Illness: A Multicenter Cluster-Randomized Trial
Source: PLoS One. 2016 May 26;11(5):e0154832. doi: 10.1371/journal.pone.0154832 (PMC4881951; doi:10.1371/journal.pone.0154832)
Supplement: S7 Table — (DOC) [file pone.0154832.s012.doc]

| S7 Table. Prescription of prophylactic treatment | | | | | | | | | | | | | | | | | | | | | |  |  |  |
| --- | --- | --- | --- | --- | --- | --- | --- | --- | --- | --- | --- | --- | --- | --- | --- | --- | --- | --- | --- | --- | --- | --- | --- | --- |
|  |  |  | Intervention group | | | | | | |  | Control group | | | | | | |  | Adjusted difference in change  (95% CI), percentage points*† | p  value |  | | | |
|  |  |  | Phase | | | | |  | Adjusted absolute change, %*§ |  | Phase | | | | |  | Adjusted absolute change, %*§ |  |  |  |  | | | |
|  |  |  | Pre-intervention | |  | Intervention | |  |  |  | Pre-intervention | |  | Intervention | |  |  |  |  |  |  | | | |
| All practice | |  |  |  |  |  |  |  |  |  |  |  |  |  |  |  |  |  |  |  |  | | | |
| Prescription of prophylactic anticoagulant treatment — no. (%) | |  | 249/712 | (35.0) |  | 4026/8359 | (48.2) |  | 9.4 |  | 281/690 | (40.7) |  | 3086/6992 | (44.1) |  | 2.9 |  | 6.5 (1.5 to 11.5) | 0.011 |  | | | |
| Treatment recommended | |  |  |  |  |  |  |  |  |  |  |  |  |  |  |  |  |  |  |  |  | | | |
| Prescription of prophylactic treatment — no. (%) | |  | 156/319 | (48.9) |  | 2669/4222 | (63.2) |  | 12.9 |  | 167/318 | (52.5) |  | 1994/3581 | (55.7) |  | 4.1 |  | 8.8 (0.8 to 16.8) | 0.030 |  | | | |
| Treatment not recommended | |  |  |  |  |  |  |  |  |  |  |  |  |  |  |  |  |  |  |  |  | | | |
| Prescription of prophylactic treatment — no. (%) | |  | 93/393 | (23.7) |  | 1357/4137 | (32.8) |  | 6.6 |  | 114/372 | (30.7) |  | 1092/3411 | (32.0) |  | 2.2 |  | 4.4 (-1.9 to 10.8) | 0.17 |  | | | |

* Adjusted for cluster effect, age, sex, history of previous thromboembolism, fracture and/or orthopedic immobilization of a lower limb within 1 month, antiplatelet therapy, main reason for admission, surgery (general or regional anesthesia), indwelling central venous catheter or cardiac stimulator implantation, length of hospitalization, number of patients admitted for year 2010 in the center.
† Difference in absolute change of adequacy between the intervention and control groups.
§ Adjusted absolute change in the frequency of adequate prevention practice between the pre-intervention and intervention periods.
