# Supplementary material for: Multifaceted Intervention to Prevent Venous Thromboembolism in Patients Hospitalized for Acute Medical Illness: A Multicenter Cluster-Randomized Trial
Source: PLoS One. 2016 May 26;11(5):e0154832. doi: 10.1371/journal.pone.0154832 (PMC4881951; doi:10.1371/journal.pone.0154832)
Supplement: S8 Table — (DOC) [file pone.0154832.s013.doc]

| | S8 Table. Three-months outcomes in the 2 hospitals for which a computerized reminder was implemented | | | | | --- | --- | --- | --- | | Thromboembolic event or major bleeding — no./N (%) | | 55/2093 | (2.6) | | First event: | |  |  | | Thromboembolic event — no./N (%) | | 29/2093 | (1.4) | |  | Pulmonary embolism (including fatal PE) — no. | 3 |  | |  | Unexplained sudden death — no. | 17 |  | |  | Proximal DVT — no. | 5 |  | |  | Distal DVT — no. | 4 |  | | Major bleeding (including fatal) — no./N (%) | | 26/2093 | (1.2) | |  | Non-fatal major bleeding — no. | 20 |  | |  | Fatal bleeding — no. | 6 |  | | Death — no./N (%) | | 230/2117 | (10.9) | |  | Fatal pulmonary embolism — no. | 0 |  | |  | Unexplained sudden death — no. | 18 |  | |  | Fatal haemorrhage — no. | 7 |  | |  | Death unrelated to PE or haemorrhage — no. | 180 |  | |  | Death with insufficient information — no. | 25 |  | |
| --- | --- | --- | --- | --- | --- | --- | --- | --- | --- | --- | --- | --- | --- | --- | --- | --- | --- | --- | --- | --- | --- | --- | --- | --- | --- | --- | --- | --- | --- | --- | --- | --- | --- | --- | --- | --- | --- | --- | --- | --- | --- | --- | --- | --- | --- | --- | --- | --- | --- | --- | --- | --- | --- | --- | --- | --- | --- | --- | --- | --- | --- | --- | --- | --- | --- | --- | --- | --- |
