# Supplementary material for: Multifaceted Intervention to Prevent Venous Thromboembolism in Patients Hospitalized for Acute Medical Illness: A Multicenter Cluster-Randomized Trial
Source: PLoS One. 2016 May 26;11(5):e0154832. doi: 10.1371/journal.pone.0154832 (PMC4881951; doi:10.1371/journal.pone.0154832)
Supplement: S9 Table — (DOC) [file pone.0154832.s014.doc]

| | S9 Table. Thromboprophylaxis practices adequacy in the 2 hospitals for which a computerized reminder was implemented | | | | | | | | --- | --- | --- | --- | --- | --- | --- | |  |  | Pre-intervention period  N = 153 | |  | Intervention period  N = 2121 | | | Adequate prevention practices — no. (%) | | 86 | (56.2) |  | 1100 | (51.9) | |  | Treatment recommended and given as recommended — no. (%) | 8 | (5.2) |  | 332 | (15.7) | |  | Treatment not recommended and not given— no. (%) | 70 | (45.8) |  | 726 | (34.2) | |  | Treatment contraindicated and not given— no. (%) | 8 | (5.2) |  | 42 | (2.0) | | Inadequate prevention practices — no. (%) | |  |  |  |  |  | |  | Treatment recommended but not given — no. (%) | 33 | (21.6) |  | 408 | (19.2) | |  | Treatment recommended, but not given as recommended — no. (%) | 21 | (13.7) |  | 320 | (15.1) | |  | Treatment not recommended (or contraindicated) but given — no. (%) | 13 | (8.5) |  | 293 | (13.8) | | Prescription of prophylactic anticoagulant treatment — no. (%) | | 42 | (27.5) |  | 945 | (44.6) | |
| --- | --- | --- | --- | --- | --- | --- | --- | --- | --- | --- | --- | --- | --- | --- | --- | --- | --- | --- | --- | --- | --- | --- | --- | --- | --- | --- | --- | --- | --- | --- | --- | --- | --- | --- | --- | --- | --- | --- | --- | --- | --- | --- | --- | --- | --- | --- | --- | --- | --- | --- | --- | --- | --- | --- | --- | --- | --- | --- | --- | --- | --- | --- | --- | --- | --- | --- | --- | --- | --- | --- | --- | --- | --- | --- | --- | --- | --- |
