# Supplementary material for: Multifaceted Intervention to Prevent Venous Thromboembolism in Patients Hospitalized for Acute Medical Illness: A Multicenter Cluster-Randomized Trial
Source: PLoS One. 2016 May 26;11(5):e0154832. doi: 10.1371/journal.pone.0154832 (PMC4881951; doi:10.1371/journal.pone.0154832)
Supplement: S10 Table — (DOC) [file pone.0154832.s015.doc]

| S10 Table. Intracluster correlation coefficients. | | | |
| --- | --- | --- | --- |
|  | Thromboembolic event or major bleeding | Death | Adequate prevention practices |
| Empty model* | 0.017 | 0.022 | 0.002 |
| Model including intervention | 0.017 | 0.023 | - |
| Model including period | - | - | 0.002 |
| Model including intervention and intervention x period | - | - | 0.001 |
| Full model† | 0.014 | 0.020 | 0.0009 |

* Model for specified outcome, including a random intercept at center level and no covariates
† Adjusted for covariates as fixed effects, according to outcome
